# An analysis of the global burden of childhood and adolescent asthma attributable to high BMI: 1990–2021

**DOI:** 10.3389/fped.2025.1646693

**Published:** 2025-10-31

**Authors:** Xinhui Zhang, Wen Wang, Wanqi Wang, Zhuiyue Wang, Anran Xu, Zheng Xue

**Affiliations:** ^1^ Department of Pediatrics, Shanghai Municipal Hospital of Traditional Chinese Medicine, Shanghai University of Traditional Chinese Medicine, Shanghai, China; ^2^ Shanghai University of Traditional Chinese Medicine, Shanghai, China; ^3^ Department of Traditional Chinese Medicine Preventive Health Care, Longhua Hospital Affiliated to Shanghai University of Traditional Chinese Medicine, Shanghai, China

**Keywords:** asthma, high BMI, childhood and adolescent, mortality, disability-adjusted life years (DALYs), Global Burden of Disease (GBD)

## Abstract

**Background:**

Childhood and adolescent obesity has become a major public health concern, contributing to the increasing burden of asthma. However, the global and regional trends of asthma attributable to high BMI in this population remain unclear. This study aimed to assess the burden of asthma due to high BMI among children and adolescents from 1990 to 2021, exploring disparities across different sex groups and Socio-Demographic Index (SDI) regions and projecting future trends.

**Methods:**

Data were extracted from the Global Burden of Disease (GBD) 2021 database. The analysis in this study covers the following indicators: age-standardized mortality rate (ASMR), age-standardized disability-adjusted life year rate (ASDR), years lived with disability (YLDs), years of life lost (YLLs), as well as crude mortality rate, crude disability-adjusted life year rate, and their respective percentage change (PC) and estimated annual percentage change (EAPC) from 1990 to 2021. Inequality in disease burden across SDI regions was quantified using the Slope Index of Inequality (SII) and Concentration Index (CI). Future burden projections were conducted using the Autoregressive Integrated Moving Average (ARIMA) model. The percentage of DALYs attributable to risk factors is reported among all age groups and individuals under 20 years.

**Results:**

From 1990 to 2021, globally, the mortality rates and numbers of asthma cases caused by high body mass index (BMI) decreased, while the numbers and rates of disability-adjusted life years (DALYs) increased. The number and rate of DALY showed different trends in SDI regions and sex groups. Statistical analysis showed a significant correlation between SDI and asthma burden (*P* < 0.001), with the overall disease burden increasing as SDI rose. However, this trend was not entirely consistent, as country variations were observed. Otherwise the higher the SDI, the lower the age-standardized mortality rate of asthma usually is. Future projections show that without effective intervention measures, the absolute burden will continue to increase.

**Conclusion:**

Despite regional differences, the global burden of asthma attributable to high BMI among children and adolescents remains substantial, with notable disparities across SDI regions. Given the projected increase in burden, urgent public health strategies targeting childhood obesity prevention and early intervention are essential to mitigate the long-term respiratory health consequences.

## 1 Introduction

Asthma is a chronic inflammatory disease characterized by reversible airflow obstruction, airway hyperresponsiveness, and recurrent respiratory symptoms such as wheezing, breathlessness, chest tightness, and coughing (1, 2). It remains a significant global health issue, particularly affecting children and adolescents, with a high disease burden in both high-income and low- and middle-income countries (3, 4). According to the Global Burden of Disease (GBD) study, asthma affected approximately 260 million people worldwide in 2019, contributing substantially to disability-adjusted life years (DALYs) and healthcare expenditures (5). Despite advancements in treatment, asthma continues to result in considerable morbidity, frequent hospitalizations, and impaired quality of life.

Asthma prevalence and severity vary across different regions, age groups, and genders. The International Study of Asthma and Allergies in Childhood (ISAAC) has reported significant geographical differences, with higher prevalence in developed and urbanized regions compared to rural areas (6, 7). In childhood, males are more commonly affected, whereas in adolescence and adulthood, asthma prevalence is higher in females, likely due to hormonal and immunological differences (8, 9). There are distinct gender differences in the association between obesity and asthma in the pediatric population (10). A systematic review and meta-analysis (16) specifically examining gender-related factors indicated a bidirectional association between obesity and asthma in children and adolescents, with childhood obesity driving an increased risk of asthma onset. The pooled relative risk (RR) for boys was 1.53 (95% confidence interval [CI]: 1.17–1.99; *p* = 0.002), whereas no such significant association was observed in girls (RR = 1.17, 95% CI: 0.79–1.72; *p* = 0.434). However, this association reverses with adolescent development: among 16–18 year-olds, the prevalence of obese asthma in females surpassed that in males, and this shift may be related to changes in sex hormone levels and differences in fat distribution.

Obese individuals exhibit significant macrophage infiltration in visceral adipose tissue. These macrophages secrete adipokines such as leptin, which may directly influence airway responsiveness (11). Altered adipokine levels, including increased leptin and decreased adiponectin, further exacerbate immune dysregulation and airway remodeling (12). Leptin secreted by adipocytes under obese conditions binds to the leptin receptor (obR) on the surface of macrophages, activates the JNK/STAT3/Akt signaling pathway, drives the polarization of macrophages toward the pro-inflammatory M1 phenotype, and releases chemokines such as CXCL2, thereby recruiting a large number of neutrophils to the airways and ultimately exacerbating obesity-related neutrophilic airway inflammation. This mechanism reveals the core role of adipokines (Leptin) and immunometabolism (M1 macrophage polarization) in obesity-asthma comorbidity (13). In the pathophysiological association between obesity and asthma, adipose tissue eosinophils, IL-4, and leptin form a key regulatory network via the immune-metabolic axis. This mechanism reveals that IL-4 deficiency caused by reduced adipose tissue eosinophils is a core node linking obesity, insulin resistance, and airway hyperresponsiveness in asthma. It provides a new direction for combined therapies targeting the leptin/IL-4 axis (e.g., the combination of anti-leptin antibodies and IL-4 receptor antagonists can reduce airway resistance in asthmatic mice by 40%) (14). Additionally, obesity-related mechanical factors, such as reduced lung compliance and increased airway closure, impair lung function and worsen asthma control (15, 16).

Additionally, asthma often coexists with allergic rhinitis, suggesting a shared underlying mechanism driven by immune dysregulation and environmental triggers (11, 21). Environmental and socioeconomic factors play a crucial role in shaping asthma burden. Exposure to air pollution, allergens, and urbanization has been linked to increased asthma prevalence and severity (17, 18). Socioeconomic disparities further exacerbate asthma outcomes, as individuals from lower-income backgrounds face greater exposure to environmental risks and reduced access to healthcare resources (19). Moreover, early-life viral infections and psychosocial stress contribute to heightened asthma susceptibility, indicating a complex interaction between genetic predisposition and environmental influences (9, 20). Understanding these multifactorial contributors is essential for developing targeted prevention and intervention strategies to reduce the global burden of asthma.

High body mass index (BMI) is a recognized risk factor for asthma, contributing to both its development and severity (17). A substantial proportion of asthma cases can be attributed to modifiable risk factors, particularly high BMI. Multiple systematic reviews and meta-analyses have confirmed a significant positive correlation between obesity and the risk of developing asthma. In this meta-analysis (16), we found a bidirectional association between asthma and obesity during childhood and adolescence. Obese children were at an increased risk of developing physician-diagnosed asthma. Obesity during childhood—a critical window for pulmonary development—disrupts the coordinated growth of airways and lung parenchyma, a phenomenon termed “airway growth dyssynapsis.” In normal development, airway caliber and lung volume expand proportionally to accommodate increasing respiratory demands. However, excess adiposity in obese children alters this harmony through mechanical compression, inflammatory dysregulation and nutrient-mediated developmental perturbations (22). In the clinical management of severe asthma, biological agents (such as anti-IgE antibodies, anti-IL-5/IL-5R antibodies, etc.) are key therapeutic approaches targeting specific inflammatory phenotypes, particularly T2 inflammation. They can significantly reduce the frequency of acute exacerbations, improve pulmonary function, and enhance quality of life. However, obese patients with severe asthma often exhibit a decreased response rate and attenuated therapeutic efficacy to these biological agents, posing a significant challenge in clinical treatment. This is because obesity primarily activates and strengthens non-T2 inflammatory pathways (which have no cross-talk or synergistic effects with T2 inflammatory pathways), leading to “inflammatory compensation” or “inflammatory superimposition” effects, thereby weakening the therapeutic efficacy of biological agents that mainly target T2 inflammatory factors (23).

Adult patients with obese asthma exhibit a more pronounced disease burden. Scott et al. found that the inflammatory profiles of obese asthma patients differ from those of non-obese asthma patients. This systematic review (24) including 40 studies showed that the proportion of neutrophils in adult asthma patients in the obese group was 5% higher than that in the non-obese group (MD = 5.0%, 95% CI: 1.2–8.9, *p* = 0.01), and the levels of serum inflammatory markers such as C-reactive protein (CRP) and interleukin-6 (IL-6) were significantly elevated. Obesity promotes systemic inflammation through elevated cytokines such as interleukin-6 (IL-6) and tumor necrosis factor-alpha (TNF-α), leading to airway inflammation and hyperresponsiveness (25). Studies have shown that increased BMI can contribute to asthma risk by inducing chronic low-grade inflammation in adipose tissue, which disrupts immune balance. Obesity-related inflammation is not confined to adipose tissue but can extend to the airways, exacerbating airway inflammation and asthma symptoms. Moreover, in individuals with obesity, immune cells within adipose tissue not only promote local inflammation but also release cytokines that influence systemic metabolic processes, further enhancing airway hyperresponsiveness (26).

Epidemiological studies indicate a dose-dependent relationship between BMI and asthma prevalence, with obesity associated with higher incidence, more severe symptoms, and poorer response to treatment (25). High BMI is a major risk factor, accounting for a substantial proportion of global asthma-related deaths and disability-adjusted life years (DALYs). In 2019, asthma contributed to over 2.15 million DALYs and 461,060 deaths worldwide (27). Yuan et al. analysed the burden of asthma by location, age, gender, and sociodemographic index (SDI) levels from 1990 to 2021, and factors such as high body mass index (BMI), occupational asthma, and smoking affect asthma globally (28). Given the increasing prevalence of childhood and adolescent obesity, addressing its role in asthma is critical for effective prevention and management strategies (29, 30). Despite the growing body of research reporting the association between high BMI and asthma risk, maintaining a normal BMI trajectory during childhood may help reduce the risk of developing asthma (31). Therefore, understanding the global burden of childhood and adolescent asthma attributable to high BMI is crucial for informing public health strategies, optimizing resource allocation, and implementing targeted interventions to mitigate the impact of obesity on respiratory health.

The Global Burden of Disease (GBD) 2021 Study provides a comprehensive dataset for assessing the burden of diseases and injuries, enabling comparisons across different regions, time periods, and sociodemographic groups. Previous research has primarily focused on the overall burden of asthma, yet the burden specifically attributable to high body mass index (BMI) remains insufficiently characterized. Building upon the extensive GBD database, this study aims to quantify the global, regional, and national burden of asthma in children and adolescents attributable to high BMI from 1990 to 2021. By analyzing temporal trends in mortality and disability-adjusted life years (DALYs), this study seeks to provide valuable insights into the evolving impact of high BMI on asthma burden in children and adolescents. Furthermore, this analysis will explore the influence of socio-demographic factors, including the Socio-Demographic Index (SDI), on the burden of asthma linked to high BMI, while also projecting future trends. The findings from this study will serve as a foundation for evidence-based public health interventions aimed at reducing the burden of asthma associated with high BMI.

## 2 Methods

### 2.1 Data source

The data for this study were obtained from the Global Burden of Disease (GBD) 2021 database, a systematic and comprehensive assessment of global, regional, and national disease burden attributable to various risk factors, including high BMI. Data were extracted via the Global Health Data Exchange (GHDx) platform, selecting “High Body Mass Index” as the risk factor, “Asthma” as the disease category, selecting “<5, 5–9, 10–14, 15–19, <20” in the age list. The analysis in this study covers the following indicators: age-standardized mortality rate (ASMR), age-standardized disability-adjusted life year rate (ASDR), years lived with disability (YLDs), years of life lost (YLLs), as well as crude mortality rate, crude disability-adjusted life year rate, and their respective percentage change (PC) and estimated annual percentage change (EAPC) from 1990 to 2021. Additionally, we retrieved Socio-Demographic Index (SDI) data to classify countries into five SDI levels, enabling a stratified analysis of disease burden across different socioeconomic contexts.

### 2.2 Definitions

High BMI was classified according to the GBD 2021 criteria for children and adolescents (ages 5–19), where BMI-for-age above the 85th percentile was defined as overweight and above the 95th percentile as obesity, based on international growth reference standards. The direct standardization method was utilized to calculate the values of the ASR for the corresponding age group (0–20 years) according to global age-standardized population weights when comparisons are made among different places (29). In the GBD 2021 study, asthma diagnosis was based on self-reported wheezing in the past 12 months and physician diagnosis (International Classification of Diseases, 9th Revision Code 493, 10th Revision Codes J45 and J46). Due to patient information desensitization in the GBD study, the Institutional Review Board of the University of Washington approved the waiver of informed consent (32). The formula is as follows: $\mathrm{ASR}=\sum_{i=1}^{A}\;a_{i}w_{i}/\sum_{i=1}^{A}\;w_{i}\times 100,000$, where ai denotes the rate of ith age group, and *w_i_* indicates the weight of the same age subgroup i in the criteria population for reference (33). Final point estimates were reported with 95% uncertainty intervals (UIs), which were 95% ranges calculated as the range from the 2.5th to the 97.5th percentile on the basis of the distribution of 1,000 draws at each GBD estimation step, with uncertainty propagated through each step.

The Socio-Demographic Index (SDI), developed by the GBD research team, was used to assess the association between asthma burden and socioeconomic development. SDI (34) is a composite measure incorporating educational attainment, income per capita, and total fertility rate among populations under 25 years old. It ranges from 0 to 1 and is classified into five levels: low (<0.46), low-middle (0.46–0.60), middle (0.61–0.69), high-middle (0.70–0.81), and high (>0.81).

### 2.3 Statistical analysis

#### 2.3.1 Burden description

The burden of asthma attributable to high BMI among children and adolescents was assessed using death rates and DALY rates (per 100,000 population) from 1990 to 2021. A positive EAPC indicates an increasing trend, while a negative EAPC suggests a decreasing trend. The 95% Uncertainty intervals (UIs) are used to assess the statistical significance of these changes. The Estimated Annual Percentage Change (EAPC) is a commonly used metric to assess the trend of Age-Standardized Rates (ASR) over a specific period, and its calculation is based on a linear regression model (33). The expression of this model is: *y* = *β x* + *α* + *ε*, where the parameters are defined as follows: *y* indicates the ln (ASR), *x* indicates the year (1990–2021), *α* indicates the intercept, *β* indicates the slope, *ε* indicates the random error (35). The specific formula for calculating EAPC is: EAPC = 100 × (exp(*β*) − 1), where “exp(*β*)” denotes taking the exponential of the regression slope *β*. Meanwhile, the model can further be used to calculate the 95% Confidence Intervals (95% CIs) for EAPC. In addition, the percentage change in rates for each age group is calculated using the following formula: Percentage Change = (Age-specific Rate in 2021—Age-specific Rate in 1990)/Age-specific Rate in 1990 (36).

#### 2.3.2 Cross-country inequality analysis

To quantify disparities in the burden of asthma attributable to high BMI across 204 countries and territories, we employed the Slope Index of Inequality (SII) and the Concentration Index (CI), both recommended by the World Health Organization (WHO). SII was used to assess absolute inequalities by measuring the difference in burden between the lowest and highest SDI groups, while CI was applied to evaluate relative disparities by fitting a Lorenz curve to the cumulative distribution of burden ranked by SDI. Higher values of SII and CI indicated greater inequality in asthma burden.

#### 2.3.3 Predictive analysis

To project future trends in asthma burden due to high BMI, we applied the Autoregressive Integrated Moving Average (ARIMA) model forecast to predict future trends in the crude DALY rate due to asthma attributable to high BMI among children and adolescents, a widely used approach for forecasting disease burden under demographic and epidemiological shifts. The model estimated crude death rates and DALY rates from 2022 to 2050, assuming no major intervention. Uncertainty in projections was quantified using 95% Uncertainty intervals (UIs). The ARIMA(3,1,1) model, comprising 3 autoregressive (AR) terms, 1 integrated (differencing, I) term, and 1 moving average (MA) term. Specifically, *p* = 3 means the model uses the previous 3 lagged values to explain the data's trend; *d* = 1 indicates the data became stationary after 1st-order differencing; *q* = 1 means the model adjusts predictions using the lagged value of the prior prediction error.

For coefficient estimates: the AR terms are ar1 = 1.5372 (s.e. = 0.2420), ar2 = −1.0566 (s.e. = 0.4117, with relatively higher uncertainty), ar3 = 0.3680 (s.e. = 0.2433); the MA term is ma1 = 0.8799 (s.e. = 0.2842). The model's residual variance (*σ*^2^) is 0.0001893, reflecting small random errors.

Key model metrics include a log-likelihood of 87.81 (higher = better fit), and information criteria AIC = −165.63, AICc = −163.23, BIC = −158.46 (lower = more optimal, confirming good model selection). On the training set, error indicators demonstrate strong performance: ME = 0.00127954 (near 0, no bias), RMSE = 0.01263661, MAE = 0.007865162, MAPE = 0.5835346 (low percentage errors), and MASE = 0.1719016 (small scaled error). The residual 1st-order autocorrelation coefficient (ACF1 = 0.03242615) is close to 0, indicating no significant residual autocorrelation and good residual independence (37–39).

#### 2.3.4 Statistical methods

All statistical analyses were conducted using R software (version 4.4.1). Time-series trends and geographic disparities were visualized using the ggplot2 package, while inequality measures were computed using the Health Equity Assessment Toolkit (HEAT) (40, 41) developed by WHO. A *p*-value < 0.05 was considered statistically significant.

## 3 Results

### 3.1 Global and regional burden of asthma attributable to high BMI

At the global level, from 1990 to 2021, the total number of crude deaths due to asthma attributable to high BMI among children and adolescents decreased from 754.77 (95% UI: 344.36, 1,233.48) to 570.11 (95% UI: 280.97, 888.49). The crude death rate declined from 0.03 (95% UI: 0.02, 0.05) per 100,000 in 1990 to 0.02 (95% UI: 0.01, 0.03) per 100,000 in 2021, with a percentage change (PC) of −0.33 (95% UI: −0.7, 0.47) and an estimated annual percentage change (EAPC) of −1.29 (95% CI: −1.37, −1.21) (Table 1). The global burden of crude disability-adjusted life years (DALYs) attributable to high BMI increased from 292,827.09 (95% UI: 135,279.86, 523,007.93) in 1990 to 415,415.72 (95% UI: 192,603.22, 744,952.52) in 2021. The crude DALY rate increased from 12.97 (95% UI: 5.99, 23.16) per 100,000 in 1990 to 15.76 (95% UI: 7.31, 28.26) per 100,000 in 2021, with a PC of 0.22 (95% UI: −0.64, 2.81) and an EAPC of 0.74 (95% CI: 0.58, 0.90) (Table 2). The total number of age-standardized death number due to asthma attributable to high BMI among children and adolescents decreased from 194.71 (95% CI: 89.02, 324.83) to 142.81 (95% CI: 69.38, 227.89). The age-standardized death rate declined from 0.03 (95% CI: 0.02, 0.06) per 100,000 in 1990 to 0.02 (95% CI: 0.01, 0.03) per 100,000 in 2021(Supplementary Table S4). The total number of age-standardized Disability-Adjusted Life Years (DALYs) number due to asthma attributable to high BMI among children and adolescents increased from 74802.23 (95% CI: 33,793.40, 40,137,020.62) to 104,881.24 (95% CI: 47,056.32, 197,319.50). The age-standardized Disability-Adjusted Life Years (DALYs) rate increased from 12.99 (95% CI: 5.87, 23.81) per 100,000 in 1990 to 15.80 (95% CI: 7.09, 29.70) per 100,000 in 2021 (Supplementary Table S5).

**Table 1:** The number and rate of childhood and adolescent asthma deaths attributable to high BMI (1990, 2021) and their percentage change (PC) and estimated annual percentage change (EAPC) (1990–2021).

Location	Sex	1990		2021		1990–2021	
		Number no. (95% UI)	Rate (per 100,000) no. (95% UI)	Number no. (95% UI)	Rate (per 100,000) no. (95% UI)	PC (rate) no. (95% UI)	EAPC (rate) no. (95% CI)
Global	Both	754.77 (344.36, 1, 233.48)	0.03 (0.02, 0.05)	570.11 (280.97, 888.49)	0.02 (0.01, 0.03)	−0.33 (−0.7, 0.47)	−1.29 (−1.37, −1.21)
Global	Female	380.49 (158.73, 675.55)	0.03 (0.01, 0.06)	312.19 (151.04, 515.05)	0.02 (0.01, 0.04)	−0.33 (−0.9, 2.08)	−0.99 (−1.07, −0.92)
Global	Male	374.28 (179.74, 601.5)	0.03 (0.02, 0.05)	257.92 (128.37, 398.42)	0.02 (0.01, 0.03)	−0.33 (−0.71, 0.48)	−1.61 (−1.71, −1.52)
High SDI	Both	67.02 (33.48, 103.24)	0.03 (0.01, 0.04)	44.77 (23.86, 66.25)	0.02 (0.01, 0.03)	−0.33 (−0.7, 0.46)	−1.26 (−1.41, −1.12)
High SDI	Female	25.7 (12.93, 39.79)	0.02 (0.01, 0.03)	20.08 (10.74, 29.79)	0.02 (0.01, 0.03)	0 (−0.56, 1.25)	−0.71 (−0.86, −0.56)
High SDI	Male	41.32 (20.27, 63.33)	0.03 (0.02, 0.05)	24.69 (13.14, 37.31)	0.02 (0.01, 0.03)	−0.33 (−0.7, 0.5)	−1.67 (−1.85, −1.49)
High-middle SDI	Both	52.19 (25.08, 81.84)	0.01 (0.01, 0.02)	19.35 (10.07, 29.48)	0.01 (0, 0.01)	0 (−0.55, 1.24)	−2.83 (−2.98, −2.68)
High-middle SDI	Female	21.68 (10.25, 36.43)	0.01 (0.01, 0.02)	9.2 (4.75, 14.16)	0.01 (0, 0.01)	0 (−0.55, 1.22)	−2.26 (−2.39, −2.13)
High-middle SDI	Male	30.5 (14.66, 47.12)	0.02 (0.01, 0.02)	10.16 (5.1, 15.79)	0.01 (0, 0.01)	−0.5 (−0.76, −0.18)	−3.28 (−3.45, −3.11)
Middle SDI	Both	197.54 (94.26, 322.4)	0.03 (0.01, 0.04)	125.22 (64.33, 192.87)	0.02 (0.01, 0.03)	−0.33 (−0.7, 0.47)	−1.1 (−1.26, −0.94)
Middle SDI	Female	100.03 (44.71, 175.82)	0.03 (0.01, 0.05)	65.38 (32.7, 104.44)	0.02 (0.01, 0.03)	−0.33 (−0.72, 1.02)	−0.93 (−1.1, −0.77)
Middle SDI	Male	97.5 (44.93, 156.31)	0.02 (0.01, 0.04)	59.84 (30.5, 94.63)	0.02 (0.01, 0.02)	0 (−0.47, 2.57)	−1.27 (−1.44, −1.1)
Low-middle SDI	Both	238.46 (109.82, 394.99)	0.04 (0.02, 0.07)	147.9 (74.47, 232.46)	0.02 (0.01, 0.03)	−0.5 (−0.79, 0.4)	−2.34 (−2.4, −2.29)
Low-middle SDI	Female	124.38 (48.71, 227.28)	0.04 (0.02, 0.08)	83.63 (40.77, 134.15)	0.02 (0.01, 0.04)	−0.5 (−0.9, 0.85)	−2.11 (−2.16, −2.06)
Low-middle SDI	Male	114.08 (54.42, 183.79)	0.04 (0.02, 0.06)	64.27 (31.78, 100.39)	0.02 (0.01, 0.03)	−0.5 (−0.77, 0.1)	−2.62 (−2.68, −2.56)
Low SDI	Both	198.48 (82.19, 357.35)	0.07 (0.03, 0.13)	231.82 (104.41, 390.22)	0.04 (0.02, 0.07)	−0.43 (−0.82, 0.99)	−1.76 (−1.86, −1.67)
Low SDI	Female	108.15 (36.78, 205.41)	0.08 (0.03, 0.15)	133.36 (59.49, 252.68)	0.05 (0.02, 0.09)	−0.38 (−0.85, 1.41)	−1.59 (−1.67, −1.51)
Low SDI	Male	90.33 (39.08, 150.37)	0.06 (0.03, 0.11)	98.47 (44.5, 164.21)	0.03 (0.01, 0.06)	−0.5 (−0.91, 0.6)	−1.97 (−2.09, −1.86)

SDI, Socio-demographic Index (a composite indicator measuring regional development levels, calculated based on three dimensions: per capita income, average years of education, and total fertility rate; higher SDI values indicate higher levels of regional development.

**Table 2:** The number and rate of disability-adjusted life years (DALYs) from childhood and adolescent asthma attributable to high BMI in 1990 and 2021, and the percentage change (PC) and the estimated annual percentage change (EAPC) from 1990 to 2021.

Location	Sex	1990		2021		1990–2021	
		Number no. (95%UI)	Rate (per 100,000) no. (95% UI)	Number no. (95% UI)	Rate (per 100,000) no. (95% UI)	PC (rate) no. (95% UI)	EAP C (rate) no. (95% CI)
Global	Both	2,92,827.09 (1,35,279.86, 5,23,007.93)	12.97 (5.99, 23.16)	4,15,415.72 (1,92,603.22, 7,44,952.52)	15.76 (7.31, 28.26)	0.22 (−0.64, 2.81)	0.74 (0.58, 0.9)
Global	Female	1,37,455.13 (63,736.26, 2,43,906.54)	12.48 (5.79, 22.15)	1,93,126.38 (89,238.4, 3,43,002.51)	15.12 (6.99, 26.85)	0.21 (−0.62, 2.84)	0.73 (0.58, 0.88)
Global	Male	1,55,371.96 (70,856.16, 2,78,430.81)	13.42 (6.12, 24.06)	2,22,289.34 (1,03,456.86, 3,97,146.24)	16.36 (7.62, 29.23)	0.22 (−0.63, 2.99)	0.75 (0.58, 0.91)
High SDI	Both	72,489.31 (33,187.77, 1,31,583.96)	28.84 (13.21, 52.36)	1,01,156.69 (47,129.58, 1,82,535.63)	43.47 (20.25, 78.43)	0.51 (−0.57, 4.02)	1.64 (1.5, 1.78)
High SDI	Female	30,802.05 (13,590.78, 54,715.38)	25.17 (11.11, 44.72)	45,455.91 (20,873.45, 1,744.38)	40.2 (18.46, 72.3)	0.6 (−0.52, 4.13)	1.93 (1.76, 2.09)
High SDI	Male	41,687.27 (19,268.09, 77,141.92)	32.33 (14.94, 59.82)	55,700.78 (26,229.39, 1,00,917.13)	46.55 (21.92, 84.34)	0.44 (−0.57, 3.93)	1.42 (1.3, 1.55)
High-middle SDI	Both	34,605.4 (15,580.08, 62,439)	9.35 (4.21, 16.87)	46,386.24 (20,087.57, 86,737.72)	15.29 (6.62, 28.59)	0.64 (−0.56, 4.61)	1.62 (1.39, 1.84)
High-middle SDI	Female	14,442.31 (6,302.03, 26,050.94)	8.03 (3.5, 14.48)	19,123.23 (8,319.6, 35,210.08)	13.24 (5.76, 24.38)	0.65 (−0.56, 4.51)	1.62 (1.37, 1.87)
High-middle SDI	Male	20,163.09 (8,968, 36,461.87)	10.59 (4.71, 19.16)	27,263 (11,804.08, 51,470.3)	17.15 (7.43, 32.38)	0.62 (−0.61, 4.45)	1.6 (1.39, 1.8)
Middle SDI	Both	83,774.14 (38,054.79, 1,49,821.01)	10.96 (4.98, 19.6)	1,09,690.29 (50,235.32, 1,96,914.44)	14.64 (6.71, 26.28)	0.34 (−0.6, 3.32)	1.06 (0.85, 1.28)
Middle SDI	Female	39,958.17 (18,008.14, 71,397.73)	10.73 (4.84, 19.18)	50,085.42 (22,531.53, 90,088.75)	13.93 (6.26, 25.05)	0.3 (−0.62, 3.29)	0.94 (0.73, 1.15)
Middle SDI	Male	43,815.97 (19,619.66, 78,831.51)	11.17 (5, 20.1)	59,604.87 (26,899.1, 1,09,153.64)	15.3 (6.91, 28.02)	0.37 (−0.63, 3.44)	1.17 (0.94, 1.39)
Low-middle SDI	Both	62,140.13 (28,445.39, 1,09,405.73)	10.51 (4.81, 18.51)	76,814.97 (35,701.72, 1,35,595.67)	10.05 (4.67, 17.74)	−0.04 (−0.71, 1.92)	−0.14 (−0.32, 0.05)
Low-middle SDI	Female	30,644.92 (13,845.36, 54,658.58)	10.63 (4.8, 18.96)	37,116.26 (17,378.58, 65,302.33)	9.98 (4.68, 17.57)	−0.06 (−0.72, 1.96)	−0.23 (−0.4, −0.06)
Low-middle SDI	Male	31,495.21 (14,089.92, 55,793.44)	10.41 (4.66, 18.43)	39,698.71 (18,157.96, 70,889.54)	10.11 (4.62, 18.05)	−0.03 (−0.7, 2.04)	−0.05 (−0.25, 0.16)
Low SDI	Both	39,439.29 (18,267.51, 68,896.85)	14.11 (6.53, 24.64)	80,838.63 (36,292.1, 1,41,040.15)	13.84 (6.21, 24.14)	−0.02 (−0.69, 1.88)	−0.09 (−0.17, −0.01)
Low SDI	Female	21,424.25 (9,668.73, 37,910.54)	15.59 (7.04, 27.59)	41,095.32 (18,527.7, 73,014.37)	14.29 (6.44, 25.4)	−0.08 (−0.73, 1.98)	−0.3 (−0.37, −0.24)
Low SDI	Male	18,015.03 (8,275.65, 31,910.12)	12.67 (5.82, 22.44)	39,743.31 (17,823.25, 69,412.01)	13.4 (6.01, 23.39)	0.06 (−0.68, 2.29)	0.15 (0.04, 0.25)

**Figure 1:**
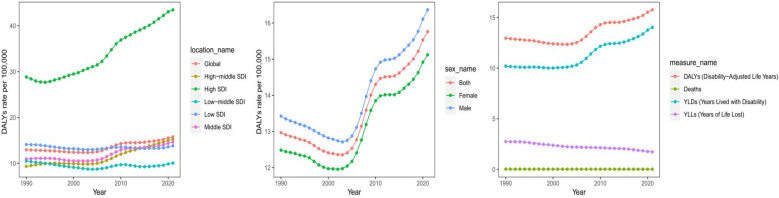
Different SDI regions burden of asthma attributable to high BMI from 1990 to 2021. DALYs, disability-adjusted life years; YLDs, years lived with disability; YLLs, years of life lost.

With regard to sex differences, in 2021, the number of asthma-related deaths due to high BMI was 257.92 (95% UI: 128.37, 398.42) among males and 312.19 (95% UI: 151.04, 515.05) among females. The EAPC of the crude death rate was −1.61 (95% CI: −1.71, −1.52) in males and −0.99 (95% CI: −1.07, −0.92) in females. The total number of DALYs was 222,289.34 (95% UI: 103,456.86, 397,146.24) in males and 193,126.38 (95% UI: 89,238.4, 343,002.51) in females. The crude DALY rate was 16.36 (95% UI: 7.62, 29.23) per 100,000 in males and 15.12 (95% UI: 6.99, 26.85) per 100,000 in females. The EAPC for crude DALY rates was 0.75 (95% CI: 0.58, 0.91) in males and 0.73 (95% CI: 0.58, 0.88) in females (Tables 1, 2, Figure 1).

In terms of SDI regions, the crude death rate significantly decreased from 1990 to 2021 in high SDI regions (EAPC = −1.26, 95% CI: −1.41 to −1.12), while the crude DALY rate increased (EAPC = 1.64, 95% CI: 1.50–1.78). High-middle SDI regions had the most substantial reduction in crude death rates (EAPC = −2.83, 95% CI: −2.98 to −2.68), whereas the crude DALY rate increased (EAPC = 1.62, 95% CI: 1.39–1.84). Middle SDI regions had a moderate decline in crude death rates (EAPC = −1.10, 95% CI: −1.26 to −0.94) and an increase in crude DALY rates (EAPC = 1.06, 95% CI: 0.85–1.28). Low-middle and low SDI regions showed the slowest declines in crude death rates (EAPC = −2.34 and −1.76, respectively), with relatively minor changes in crude DALY rates (EAPC = −0.14 in low-middle SDI regions and −0.09 in low SDI regions) (Tables 1, 2, Figure 1).

### 3.2 National burden of asthma attributable to high BMI

**Figure 2:**
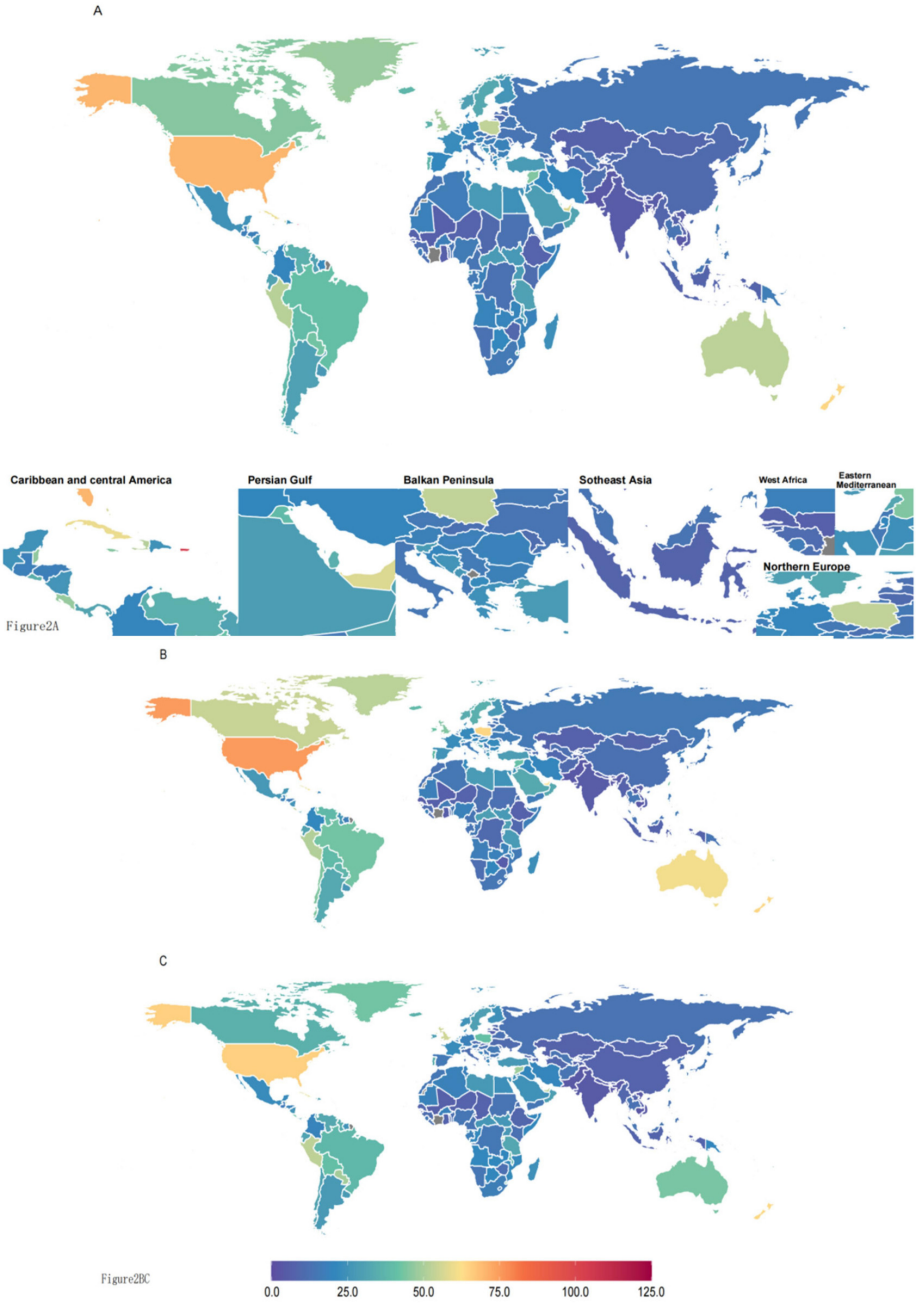
**(A)** Both sexes° **(B)** males° **(C)** females. DALYs (disability-adjusted life years) quantify the total health loss from both fatal and non-fatal outcomes; one DALY equals one lost year of healthy life. All rates are reported per 100,000 individuals aged <20 years.

In 2021, the burden of asthma attributable to high BMI varied considerably across countries (Supplementary Table S1, Figure 2). In 2021, the highest DALYs attributable to high BMI for asthma were observed in Puerto Rico, reaching 103.99 (95% UI: 47.3–191.86). In contrast, Bangladesh had the lowest DALYs, at 3.09 (95% UI: 1.25–5.97). Among countries with the highest asthma-related DALYs due to high BMI, Puerto Rico, United States Virgin Islands, Bermuda, and the United States of America ranked in the top four, with DALYs of 103.99 (95% UI: 47.3–191.86), 78.23 (95% UI: 34.58–140.57), 70.86 (95% UI: 32.47–129.21), and 70.51 (95% UI: 33.37–125.83), respectively. Conversely, Cambodia, Pakistan, and Bangladesh ranked in the bottom three, with DALYs of 3.54 (95% UI: 1.63–6.33), 3.54 (95% UI: 1.59–6.32), and 3.09 (95% UI: 1.25–5.97), respectively.

### 3.3 Burden of asthma attributable to high BMI by age and sex

Globally, the burden of asthma attributable to high BMI in 2021 varied across age groups (Supplementary Figure F1), among females, with the highest DALY count observed in the 5–9 years group (58,456), followed by the10–14 years (52,433), <5 years (43,629), and 15–19 years groups (38,609). Among males, the highest DALY count was recorded in the 5–9 years group (71,507), followed by the10–14 years (61,096), <5 years (57,618), and 15–19 years groups (32,068). In 2021, the burden of asthma attributable to high BMI varied considerably across sex groups, the disease burden in males was higher than in females.

### 3.4 Burden of asthma attributable to high BMI associated with SDI

In 2021, the Years of Life Lost (YLLs) due to asthma attributable to high BMI was highest in low SDI regions, where females (3.77, 95% UI: 1.67–7.10) and males (2.75, 95% UI: 1.24–4.60) experienced the greatest premature mortality. The burden decreased with increasing SDI, with low-middle SDI and middle SDI regions showing progressively lower YLL rates. In high SDI regions, the YLL burden was moderate (both: 1.48, 95% UI: 0.79–2.19), while high-middle SDI regions had the lowest YLL rates, with both sexes below 0.50 per 100,000. The asthma-related mortality rate due to high BMI followed a similar trend, being highest in low SDI regions. In high SDI regions, both sexes had a mortality rate of 0.02 (95% UI: 0.01–0.03) per 100,000, while high-middle SDI regions had the lowest mortality rates, at 0.01 (95% UI: 0–0.01) for both sexes.

In terms of DALYs, the asthma burden was highest in high-SDI regions, with males (46.55, 95% UI: 21.92–84.34) and females (40.20, 95% UI: 18.46–72.30) experiencing the greatest disease impact. In contrast, the lowest DALY burden was observed in low-middle SDI regions, where males (10.11, 95% UI: 4.6–18.05) and females (9.98, 95% UI: 4.68–17.57) were least affected (Supplementary Table S2).

The figure (Supplementary Figure F2) depicts the relationship between the age-standardized DALY rate (Disability-Adjusted Life Years per 100,000 population) and SDI. The trend line indicates a positive correlation (reported *r* = 0.372, *p* < 0.001). Statistical analysis showed a significant correlation between SDI and asthma burden (*P* < 0.001), with the overall disease burden increasing as SDI rose. However, this trend was not entirely consistent, as country variations were observed. For countries with higher SDI, ASDR does not increase linearly; instead, it exhibits the characteristic that “ASDR is relatively high in the middle SDI range, while it is relatively divergent in the high and low SDI ranges.” Some high-SDI regions in the Caribbean, such as Puerto Rico (SDI ≈ 0.85, ASDR ≈ 100 per 100,000 population) and the U.S. Virgin Islands (SDI ≈ 0.8, ASDR ≈ 70 per 100,000 population), have relatively high ASDR. Meanwhile, low-SDI countries in Sub-Saharan Africa, such as Somalia and South Sudan, also have high ASDR.

The figure (Supplementary Figure F3) depicts the relationship between the age-standardized Death rate (per 100,000 population) and SDI. The trend line indicates a positive correlation (reported *r* = −0.614, *p* < 0.001). It indicates a significant moderately strong negative correlation between ASMR (Age-Standardized Mortality Rate of Asthma) and SDI (Socio-Demographic Index). The higher the SDI, the lower the age-standardized mortality rate of asthma usually is.

Countries with low SDI (such as Somalia, Chad, Mali in Sub-Saharan Africa, etc.) fall into the lower SDI range (the left side of the horizontal axis), yet their ASMR is relatively high. Countries with high SDI (such as Germany, Japan, Norway, Australia, etc.) lie in the higher SDI range (the right side of the horizontal axis), and their ASMR is close to 0. For regions with medium-to-high SDI but relatively high ASMR, the Pacific island nations in the upper right corner of the figure (such as Niue, Tokelau, Kiribati, Nauru, etc.) have medium-to-high SDI, but their ASMR is significantly higher than that of other high-SDI countries.

### 3.5 Cross-country inequality analysis of asthma attributable to high BMI

In 2021, significant disparities were observed in the burden of asthma attributable to high BMI across countries. The slope index of inequality (SII) was 15.18 in 2021, compared to 3.53 in 1990 (Supplementary Figure F4).

The concentration index of lifetime risk of asthma attributable to high BMI remained positive from 1990 to 2021, indicating that the disease burden was consistently concentrated in regions with higher socioeconomic development. However, over the 30-year period, this index showed an overall declining trend among males, decreasing from 0.267 (95% CI: 0.245, 0.290) in 1990 to 0.236 (95% CI: 0.214, 0.258) in 2021. In contrast, among females, the index exhibited a gradual upward trend, increasing from 0.195 (95% CI: 0.173, 0.218) in 1990 to 0.234 (95% CI: 0.213, 0.255) in 2021. These findings suggest that socioeconomic-related health inequalities in obesity-induced asthma have generally decreased among boys but increased among girls (Supplementary Figure F5, Supplementary Table S3).

### 3.6 Prediction analysis of asthma burden attributable to high BMI

The Autoregressive Integrated Moving Average (ARIMA) model forecast was used to predict future trends in the Age−Standardized DALYs (Disability−Adjusted Life Years) Rate due to asthma attributable to high BMI among children and adolescents (Supplementary Figure F6, Supplementary Table S6).

As shown in, the global burden is projected to continue rising over the next decades. By 2030, the Age-Standardized DALYs (Disability-Adjusted Life Years) Rate per 100,000 population will reach 16.88 (95% CI: 13.41–20.84), increasing to 17.6 (95% CI: 15.75–16.32) by 2036.

Sex-stratified projections indicate a persistent disparity, with males expected to have a higher DALY rate than females. By 2030, the Age−Standardized DALYs rate will be 16.74 (95% CI: 12.8–20.82) for males and 15.34 (95% CI: 11.39–19.41) for females. By 2036, the values are projected to reach 17.60 (95% CI: 15.75–16.32) for males and 15.53 (95% CI:14.9–15.47) for females.

### 3.7 DALYs attributable to risk factors (%) among all ages and <20 years between male and female

At the global level, the proportions of high BMI-related asthma DALYs among adolescent males and females are relatively close (7.6% vs. 7.5%). However, in China, the proportion among males (8.3%) is 1.9 percentage points higher than that among females (6.4%) (95% CI: 1.2–2.6). This discrepancy is primarily attributed to the fact that the obesity rate among Chinese male adolescents (21.5%) is 2.8 percentage points higher than the global male average (18.7%) (WHO 2024), while the obesity rate among Chinese female adolescents (16.3%) is roughly equivalent to the global female average (16.1%), indicating that Chinese male adolescents face a more prominent risk of high BMI exposure.

The proportion of high BMI-related asthma DALYs among Chinese female adolescents (6.4%) is lower than that among males (8.3%) and also lower than the global female average (7.6%), with the corresponding obesity rate among female adolescents (16.3%) being only 75.8% of that among males (Supplementary Figure F7).

## 4 Discussion

The study presents a comprehensive summary of the burden of asthma in children and adolescents attributable to high BMI at global, regional, and national levels, and projects future trends based on the latest GBD 2021 data. This analysis aims to provide policymakers with insights for effective resource allocation and targeted prevention strategies (42).

Based on data from the Global Burden of Disease (GBD) Study, the burden of age—standardized disability—adjusted life years rate (ASDR) of asthma exhibits a “socio—economic differentiation” worldwide: In countries with a higher Socio—Demographic Index (SDI), ASDR does not increase linearly. Instead, it shows the characteristic that “ASDR is relatively high in the medium SDI range, while it is relatively differentiated in the high and low SDI ranges”. This is consistent with the mechanism that “regions with high SDI have high diagnosis rates but good prognosis, while regions with low SDI have low diagnosis rates but poor prognosis”.

High—SDI countries show “high prevalence but low disease burden”, while low—SDI countries show “low prevalence but high disease burden”. The increasing burden in some high—SDI countries suggests that attention should be paid to controllable risk factors such as obesity and the environment. The reasons are as follows: Regions with high SDI have sufficient medical resources, leading to a high diagnosis rate of asthma, and standardized treatment can effectively reduce the risks of death and disability (thus lowering ASDR). In regions with low SDI, access to medical care is poor, resulting in a high rate of missed diagnosis of asthma, and patients often see a sharp increase in the risk of death/disability due to comorbidities (such as infections, malnutrition) or delayed treatment (thus pushing up ASDR).

Some high—SDI countries (such as Poland and the United States) experienced a continuous increase in asthma burden [including age—standardized prevalence rate (ASPR) and ASDR] from 1990 to 2021, which is contrary to the declining trend of disease burden in high—SDI regions globally. This may be related to factors such as the rising obesity rate (high BMI is a major risk factor for asthma) and changes in environmental exposure (such as occupational allergens, air pollution).

Regarding the differences in asthma mortality burden among countries with different SDI levels: Low—SDI countries (such as Somalia, Chad, and Mali in Sub—Saharan Africa) have lagging socio—economic development and scarce medical resources (insufficient diagnostic capabilities, lack of therapeutic drugs/standardized management). Asthma patients in these countries are prone to death due to acute attacks, comorbid infections, or lack of effective intervention, so the “asthma disease burden in terms of mortality” is heavier. High—SDI countries have well—established medical systems and strong capabilities in the early diagnosis of asthma, long—term standardized treatment (such as the accessibility of inhaled medications), and whole—course management. These capabilities can effectively control the disease and reduce the risk of death, so the asthma—related mortality rate is significantly low, and the mortality burden is extremely light.

Pacific island countries (such as Niue, Tokelau, Kiribati, and Nauru) are affected by specific factors such as genetic susceptibility, special allergens/environmental triggers under tropical climates (such as biological stimuli), or the unequal “accessibility/quality of medical resources”. Their asthma mortality risk does not decrease synchronously with the increase in SDI, making them “exceptions” among high—SDI groups.

Overall, the level of socio—economic development is a key influencing factor of the asthma mortality burden (the higher the SDI, the lower the asthma mortality rate). However, in some special regions such as certain Pacific island countries, there are also specific factors related to geography, environment, or medical systems, which cause their asthma mortality burden to deviate from the overall negative correlation trend between SDI and the age—standardized mortality rate of asthma (ASMR).

The results of this study highlight the significant global burden of asthma attributable to high BMI among children and adolescents, with notable variations across different sociodemographic index (SDI) regions. While the disability-adjusted life years (DALYs) increased substantially from 1990 to 2021, the corresponding death rate exhibited a declining trend. These findings suggest that despite improvements in healthcare and asthma management, the rising prevalence of childhood obesity continues to drive an increasing absolute disease burden (25, 43).

The reasons behind this phenomenon lie in the following: High SDI regions generally allocate 10%–18% of their GDP to healthcare (e.g., 17.8% in the United States and 12.3% in Germany), with approximately 5%–8% of this investment directed toward obesity-related specialties such as metabolic surgery and nutrition (44). In contrast, low SDI regions typically spend less than 5% of their GDP on healthcare (e.g., 3.5% in sub-Saharan Africa), with priority given to infectious diseases like malaria and tuberculosis. Obesity treatment is classified as a “non-urgent need,” resulting in a shortage of specialized physicians at a ratio of 1 per 100,000 people (compared to 1 per 10,000 in high SDI regions).

High SDI regions have achieved multi-level management of obesity through technological innovation, policy coordination, and resource concentration, but face issues such as drug abuse, excessive surgeries, and widening socioeconomic disparities. Low SDI regions, constrained by resource scarcity, data gaps, and cultural conflicts, rely primarily on basic interventions with limited effectiveness.

To bridge the global gap in obesity treatment, future efforts should focus on international cooperation (e.g., drug patent sharing), capacity building in primary healthcare, and the development of localized strategies. For example, the WHO recommends that low- and middle-income countries prioritize the promotion of lifestyle interventions and generic drugs, and leverage mobile health to expand access to health education. For adolescent populations, low SDI regions could utilize “school education systems” to implement early interventions. Additionally, incorporating “obesity treatment” into the “priority list for chronic disease prevention and control” in low SDI regions can indirectly reduce the burden of asthma, diabetes, and other diseases by lowering obesity rates. For pediatrics, we should take action to deliver early preventive education on childhood obesity and asthma, conduct long-term follow-up and monitoring, and guide patients and families in implementing interventions.

To clearly understand why obesity exerts an impact on the severity of asthma and the risk of acute exacerbations, it is first necessary to clarify the physiological mechanism of interaction between the two. Obesity drives Th2-type (eosinophilic) or Th17-type (neutrophilic) inflammation in asthma through an imbalance between pro-inflammatory factors (leptin, TNF-α, IL-6) and anti-inflammatory factors (adiponectin) secreted by adipose tissue. The effectiveness of obesity treatment directly determines whether this inflammatory imbalance can be corrected, thereby influencing the severity of asthma and the risk of acute exacerbations (45, 46, 54).

The core of comorbidity between obesity and asthma lies in the interaction between insulin resistance (IR) and metabolic remodeling of immune cells. Whether obesity treatment can improve IR directly determines the function of pulmonary immune cells (macrophages, T cells), which in turn affects the treatment response and prognosis of asthma (14).

On a physical level, obesity directly impairs lung function through mechanical compression caused by adipose tissue accumulation in the chest wall and airway remodeling. Whether obesity treatment can reduce body fat load directly influences airway patency and reversibility, thereby affecting the severity of asthma symptoms and the rate of decline in lung function.

Our study observed higher Disability-Adjusted Life Year (DALY) rates in males during childhood/adolescence. Gender disparities were also evident, with males consistently showing higher DALY rates and mortality rates compared to females across most SDI regions (10, 47). These differences may be influenced by sex-based variations in lung development, airway inflammation, and hormonal regulation, which have been implicated in previous research as potential contributors to differential asthma susceptibility between boys and girls (8, 9). However, the observed gender gap varied across regions, indicating that sociocultural and environmental factors may also play a role in shaping asthma outcomes.

From a physiological mechanism perspective, estrogen exerts a direct protective effect on asthma by inhibiting Th2-type inflammatory responses (e.g., reducing the release of IL-4, IL-5, and IL-13) and lowering airway hyperresponsiveness (48). Studies have found that the expression of estrogen receptor *α* (ER*α*) is significantly elevated in airway epithelial cells of female asthma patients, and targeted knockdown of ER*α* can block the ferroptosis-airway epithelial-mesenchymal transition pathway, thereby alleviating airway remodeling. In contrast, testosterone may promote visceral fat accumulation and the release of pro-inflammatory factors. The elevated estrogen levels in female adolescents may weaken the driving effect of obesity on asthma (55).

Sex hormones drive gender differences through immune regulation and airway physiological remodeling. Estrogen can activate immune genes such as TLR7/TLR8 on the X chromosome, enhance eosinophil infiltration, and promote IL-13-mediated mucus secretion, making female asthma patients more prone to hormone-dependent acute exacerbations (e.g., during menstrual periods or perimenopause). In contrast, testosterone inhibits the airway epithelial-mesenchymal transition pathway, reduces airway smooth muscle hyperplasia, and slows the progression of asthma in males. Notably, this regulation is not absolute—obesity can reverse hormonal effects. For example, in obese women, the protective effect of testosterone is attenuated by metabolic inflammation, while in lean men, estradiol can help inhibit airway hyperresponsiveness (48).

After remission of childhood asthma, pubertal hormonal changes act as a key “switch” for recurrence: In males, the elevation of testosterone during puberty suppresses Th2 inflammation, leading to a decreasing risk of recurrence with age (a 5.7% recurrence rate among 18–25-year-olds). In females, the pubertal surge in estrogen activates latent airway inflammatory pathways; even in asymptomatic cases, fractional exhaled nitric oxide (FeNO) remains elevated, resulting in a 19.3% recurrence rate by age 30. Additionally, male patients with high childhood BMI face a 2.3-fold higher risk of asthma recurrence in adulthood compared to those with normal weight, due to testosterone's insufficient regulation of visceral fat. In females, however, this association is weaker, attributed to estrogen's inhibitory effect on adipose inflammation (49).

In other studies, the burden of asthma due to high BMI in adolescents and young adults (AYA, ages 15–39) is similar to this study in that the burden of asthma DALY is higher in areas with high SDI and the burden of asthma deaths due to high BMI is higher in areas with low SDI, with the difference that the burden of DALYs is higher in women than in men in adolescents and young adults, and higher in men than in children and adolescents (50).

In the projected global burden of asthma in children and adolescents to 2050 study, the prevalence of asthma is projected to continue to decline through 2050, but remains a significant health burden for children and adolescents, consistent with the projections of DALY burden projected in this study, the global burden of DALY will continue to rise in the coming decades, and the projections by sex show that the burden will continue to be higher in men than in women (51).

The findings of this study underscore the importance of tailored public health strategies to address the growing burden of asthma attributable to high BMI. In high-SDI regions, priority should be given to comprehensive obesity prevention programs, incorporating nutritional education, physical activity promotion, and early lifestyle interventions to curb rising obesity rates among children and adolescents (52, 53). In contrast, low-SDI regions require enhanced healthcare accessibility, improved diagnostic capabilities, and community-based intervention programs to bridge existing disparities in asthma management and prevention (53). Given the projected future increase in burden, a global effort integrating multi-sectoral collaboration and policy-driven interventions is essential to mitigate the long-term impact of high BMI on childhood respiratory health.

This study has several limitations. The GBD 2021 dataset, while comprehensive, may have limited data availability for childhood and adolescent asthma in low-SDI regions, potentially underestimating the true burden. Additionally, the analysis did not distinguish between different BMI subtypes, which may have varying effects on asthma risk, restricting a more nuanced understanding of obesity-related respiratory impacts. Future projections are also subject to uncertainty, as shifts in obesity prevalence, advancements in healthcare, and the implementation of public health interventions could influence the actual disease burden over time.

## 5 Conclusion

Globally, the death and DALY rates of asthma attributable to high BMI among children and adolescents have shown varying trends across different SDI regions and sex groups from 1990 to 2021. Future projections suggest a continued increase in the absolute burden, highlighting the urgent need for targeted obesity prevention strategies and early intervention measures to mitigate the long-term impact of high BMI on respiratory health in children and adolescents.

## Data Availability

The original contributions presented in the study are included in the article/Supplementary Material, further inquiries can be directed to the corresponding author.
