# Supplementary material for: An analysis of the global burden of childhood and adolescent asthma attributable to high BMI: 1990–2021
Source: Front Pediatr. 2025 Oct 31;13:1646693. doi: 10.3389/fped.2025.1646693 (PMC12615406; doi:10.3389/fped.2025.1646693)
Supplement: Supplementary file 1 [file Datasheet1.pdf]

Supplementary Table S1 Rate (per100, 000) of DALYs among children and adolescents with asthma caused by high BMI in 204 countries by sex in 2021.

Location	BothNo. (95%UI)	MaleNo. (95%UI)	FemaleNo. (95%UI)
Puerto Rico	103.99 (47.3,191.86)	116.04 (53.41,218)	91.39 (40.05,163.71)
United States Virgin Islands	78.23 (34.58,140.57)	83.05 (35.82,154.59)	73.12 (32.73,133.82)
Bermuda	70.86 (32.47,129.21)	77.23 (33.92,139.54)	64.29 (29.12,120.93)
United States of America	70.51 (33.37,125.83)	75.22 (34.79,132.79)	65.58 (30.48,119.67)
New Zealand	64.27 (30.43,117.72)	64.46 (30.51,118.37)	64.07 (30.2,118.08)
Dominica	63.51 (29.79,108.28)	60.47 (27.56,106.67)	66.68 (30.73,118.58)
Barbados	60.32 (25.57,107.8)	51.71 (21.51,94.3)	69.37 (30.9,127.07)
Cuba	59.63 (26.33,111.85)	60.28 (25.32,115.32)	58.95 (26.32,109.38)
United Arab Emirates	57.16 (25.36,100.72)	62.6 (28.06,110.01)	51.44 (22.25,92.54)
Bahamas	56 (25.4,103.98)	54.77 (24.14,101.42)	57.24 (25.95,107.69)
Poland	53.03 (22.56,97.35)	64.39 (27.82,118.02)	41.06 (16.08,76.68)
Australia	52.42 (24.84,98.05)	60.66 (26.52,113.62)	43.71 (19.75,81.69)
Peru	51.72 (22.47,95.87)	50.96 (21.35,96.44)	52.54 (22.52,99.5)
Grenada	51.68 (22.55,98.04)	50.09 (22.18,97.27)	53.39 (22.74,99.4)
Haiti	51.23 (21.78,90.14)	56.96 (25.02,100.71)	45.4 (19.13,83.97)
United Kingdom	49.7 (22.59,89.23)	44.35 (20.01,81.73)	55.29 (25.03,96.97)
Saint Kitts and Nevis	49.67 (22.48,90.76)	51.02 (21.82,96.71)	48.3 (22.43,89.49)
Greenland	47.56 (21.65,88.16)	52.06 (23.46,94.71)	42.82 (19.4,81)
Niue	47.52 (25.57,73.27)	45.02 (23,71.02)	50.33 (25.54,83.39)
Costa Rica	47.17 (20.24,88.35)	51.57 (22.74,97.52)	42.65 (17.67,81.12)
Antigua and Barbuda	47.12 (20.64,85.03)	46.29 (19.67,84.21)	47.98 (21.11,88.22)
Belize	46.53 (20.66,83.18)	44.29 (19.33,78.85)	48.79 (21.1,90.33)
Canada	45.14 (19.78,84.75)	54.07 (24.73,102.35)	35.7 (14.64,69.84)
Jamaica	45.03 (19.88,82.93)	37 (15.73,69.35)	53.34 (23.36,97.05)
Tokelau	44.93 (23.2,73.65)	43.35 (21.14,76.93)	46.7 (22.39,83.43)
Syrian Arab Republic	44.51 (22.84,73.08)	40.83 (20.59,68.06)	48.26 (23.81,85.92)
Paraguay	42.92 (18.64,81.27)	37.11 (16.18,73.35)	49.03 (20.88,90.4)
Rwanda	42.42 (19.17,78.67)	32.82 (14.36,60.09)	52.08 (23.5,99.48)
Comoros	42.38 (20.57,71.9)	39.47 (18.17,67.62)	45.38 (21.58,79.15)
Kiribati	41.38 (21.44,66.41)	45.22 (23.36,79.43)	37.37 (18.32,61.52)
Brazil	40.59 (18.34,77.2)	42.99 (19.03,80.62)	38.1 (16.36,71.63)
Bolivia (Plurinational State of)	39.64 (18.36,69.4)	38.79 (17.97,68.92)	40.53 (18.45,71.93)
Kuwait	39.26 (17.14,71.56)	46.39 (20.66,84.32)	31.72 (13.9,58.91)
Tonga	38.99 (19.57,66.83)	40.2 (20.11,69.76)	37.68 (18.19,66.27)
Eswatini	38.47 (17.53,66.9)	33.64 (15.52,58.77)	43.59 (19.91,79.06)
Chile	37.98 (16.13,72.12)	44.46 (18.58,85.78)	31.19 (13.05,57.54)
Iceland	37.43 (16.44,69.07)	38.92 (16.29,74.09)	35.87 (14.99,66.97)
Portugal	36.81 (16.22,69.96)	38.38 (16.99,72.02)	35.17 (15.72,66.44)
Nauru	36.69 (19.16,56.99)	39.72 (20.39,62.9)	33.41 (16.61,54.08)

Location	BothNo. (95%UI)	MaleNo. (95%UI)	FemaleNo. (95%UI)
Ireland	35.82 (15.32,65.46)	41.88 (17.97,76.87)	29.48 (12.39,55)
Venezuela (Bolivarian Republic of)	35.5 (15.59,65.96)	38.5 (16.74,70.24)	32.42 (14.16,62.96)
Qatar	35.26 (15.81,66.17)	35.53 (15.52,65.78)	34.98 (15.56,64.06)
El Salvador	34.98 (15.45,65.84)	33.76 (14.4,65.85)	36.27 (15.78,65.79)
Taiwan (Province of China)	34.25 (14.69,68.29)	43.26 (18.37,85.57)	24.49 (9.97,50.01)
Palau	33.84 (17.28,56.15)	30.51 (15.01,50.62)	37.41 (19.33,63.91)
Sweden	32.54 (13.58,64.22)	35.27 (14.53,73.94)	29.63 (12.24,54.92)
Bahrain	32.07 (14.8,56.58)	30.84 (14,54.79)	33.45 (14.96,61.2)
Lebanon	31.7 (13.88,57.54)	36.27 (16.15,67.01)	26.68 (11.65,49.93)
Panama	31.54 (13.9,56.83)	33.18 (14.41,63.12)	29.82 (12.92,53.63)
Saint Lucia	31.4 (14.4,55.5)	29.47 (13.04,53.06)	33.44 (14.66,61.49)
Cook Islands	31.39 (14.63,55.43)	30.79 (14.04,54.61)	32 (14.69,57.48)
Oman	30.91 (13.48,58.02)	32.63 (13.52,63.43)	29.11 (12.97,53.36)
Ecuador	30.87 (13.69,56.22)	30.63 (13.6,57.19)	31.12 (13.52,56.87)
Monaco	30.73 (13.31,58.61)	34.55 (14.77,64.02)	26.83 (11.08,52.19)
Guyana	30.45 (13.48,54.04)	26.53 (12.06,48.2)	34.5 (15.54,62.49)
Saint Vincent and the Grenadines	30.41 (13.1,55.57)	26.51 (11.3,48.61)	34.46 (14.94,64.14)
Argentina	30.17 (13.17,58.43)	32.78 (14.48,64.85)	27.47 (11.46,53.12)
United Republic of Tanzania	29.97 (12.82,57.28)	27.8 (11.68,53.09)	32.13 (14.15,60.14)
Norway	29.66 (13.13,57.06)	34.09 (15,67.19)	24.99 (10.62,46.98)
Equatorial Guinea	29.37 (14.59,50.45)	30.67 (14.63,53.69)	27.7 (13.54,47.51)
Malawi	29.36 (13.74,50.82)	21.09 (10.02,37.54)	37.52 (17.47,63.9)
Saudi Arabia	28.91 (13.16,52.91)	32.89 (14.52,62.45)	24.38 (11.77,43.21)
San Marino	28.8 (12.77,52.32)	31.71 (13.9,58.7)	25.65 (10.79,46.31)
Uganda	28.12 (12.73,49.49)	29.07 (13.25,52.14)	27.13 (11.24,49.16)
Turkey	27.82 (12.23,53.08)	28.6 (12.61,54.28)	27 (11.89,48.47)
Central African Republic	27.71 (12.37,46.88)	29.17 (12.96,50.9)	26.23 (10.84,47.41)
Andorra	27.68 (12.73,50.99)	29.51 (13.21,56.97)	25.76 (11.19,47.76)
Slovenia	27.68 (11.68,52.89)	35.18 (15.07,66.26)	19.71 (8.05,39.01)
Libya	27.37 (12.39,47.26)	27.73 (12.62,48.69)	26.99 (12.3,47.2)
Northern Mariana Islands	27.24 (12.93,47.4)	28.28 (13.1,48.68)	26.12 (12.52,47.5)
Cyprus	27.23 (11.12,53.56)	28.59 (12.06,57.29)	25.77 (10.02,50.01)
Uruguay	27.16 (12.03,53.66)	26.45 (11.31,53.28)	27.9 (12.68,54.52)
Trinidad and Tobago	27.01 (12.43,46.29)	29.61 (13.28,51.87)	24.32 (10.26,43.33)
South Sudan	26.87 (12.1,48.05)	27.39 (11.79,51.5)	26.31 (10.92,49.88)
Luxembourg	26.86 (11.28,49.34)	36.5 (15.51,68.86)	16.58 (6.9,31.72)
Fiji	26.74 (13.04,43.56)	25.81 (12.12,43.23)	27.72 (13.79,43.67)
Samoa	26.4 (12.8,45.29)	22.81 (11.04,38.3)	30.24 (14.98,53.95)
Montenegro	26.1 (10.9,49.54)	31.45 (13.49,60.09)	20.29 (8.48,39.01)
Finland	25.76 (11.37,50.82)	27.11 (11.83,51.23)	24.34 (10.26,46.98)

Location	BothNo. (95%UI)	MaleNo. (95%UI)	FemaleNo. (95%UI)
Nicaragua	25.69 (11.14,47.37)	25.6 (11.26,50.51)	25.79 (10.5,46.13)
American Samoa	25.44 (12.46,43.31)	25.02 (11.99,42.63)	25.88 (12.7,42.49)
Bosnia and Herzegovina	25.15 (10.52,47.43)	30.91 (12.89,59.45)	19.05 (7.59,35.86)
Marshall Islands	24.75 (12.53,39.97)	23.83 (11.47,39.44)	25.72 (13.16,41.32)
Mexico	24.67 (11.03,44.83)	26.9 (11.97,49.27)	22.39 (9.63,41.87)
Dominican Republic	24.44 (11.21,43.43)	19.11 (8.81,33.11)	29.95 (13.37,54.67)
Micronesia (Federated States of)	24.37 (12.1,40.63)	24.38 (12.17,41.14)	24.36 (12.35,39.19)
Egypt	24.31 (11.4,42.88)	24.77 (11.68,43.85)	23.82 (11.16,42.15)
Greece	24.01 (10.77,46.26)	28.37 (12.48,56.58)	19.45 (8.27,38.32)
Guam	24 (11.31,43.5)	27.42 (13.31,47.53)	20.35 (9.2,38.15)
Jordan	23.79 (10.73,42.75)	24.21 (10.85,45.47)	23.34 (10.51,41.76)
Madagascar	23.78 (10.84,40.57)	23.55 (9.89,42.21)	24.01 (9.93,44.34)
Tuvalu	23.56 (11.72,39.26)	25.28 (12.29,43.33)	21.62 (10.77,36.05)
Denmark	22.7 (9.98,41.95)	23.79 (10.54,44.28)	21.55 (9.14,39.85)
Malta	22.62 (9.54,42.31)	23.84 (10.39,46.06)	21.3 (8.43,41.21)
France	22.42 (9.65,43.13)	22.57 (9.67,45.44)	22.26 (9.31,41.94)
North Macedonia	22.07 (9.21,42.69)	24.93 (10.41,49.95)	19.02 (7.74,35.89)
Djibouti	21.95 (9.84,38.33)	19.55 (8.67,35.41)	24.81 (11.01,44.05)
Netherlands	21.59 (9.91,40.92)	19.41 (8.5,37.56)	23.88 (10.22,44.82)
Suriname	21.22 (9.02,40.07)	17.86 (7.4,34.07)	24.78 (10,45.08)
Switzerland	21.1 (8.87,40.42)	24.78 (10.46,47.68)	17.19 (7.13,32.7)
Iraq	20.86 (9.31,37)	21.32 (9.39,39.08)	20.38 (8.58,35.57)
Spain	20.69 (9.01,40.29)	25.43 (11.05,48.71)	15.67 (6.46,31.09)
Botswana	20.68 (9.68,36.26)	20.56 (9.75,35.69)	20.81 (9.4,35.65)
Palestine	20.59 (8.85,37.69)	21.13 (8.86,40.56)	20.02 (8.3,37.36)
Tunisia	20.28 (8.65,38.56)	21.93 (9.42,42.21)	18.48 (7.97,35.42)
Iran (Islamic Republic of)	20.26 (8.98,38.14)	20.26 (8.76,39.22)	20.25 (9.09,37.57)
Croatia	20.23 (8.46,38.85)	26.55 (10.78,50.79)	13.54 (5.59,26.76)
Colombia	20.15 (8.23,37.32)	21.07 (8.73,39.8)	19.18 (7.79,34.49)
Germany	20.06 (8.71,37.32)	21.8 (9.35,40.5)	18.2 (7.89,35.23)
Zambia	19.69 (9.5,33.24)	18.58 (8.54,32.31)	20.78 (9.68,36.61)
Angola	19.25 (8.87,33.38)	17.45 (8.04,30.64)	21.02 (9.78,38.56)
Somalia	19.09 (8.48,35.55)	17.58 (7.69,32.06)	20.72 (8.98,38.94)
Romania	18.85 (8.15,37.59)	21.85 (8.92,45.38)	15.67 (6.68,31.38)
Mozambique	18.83 (8.49,34.48)	15.92 (7.3,28.24)	21.68 (9.76,42.05)
Mauritania	18.36 (8.11,34)	17.97 (7.57,34.96)	18.76 (8.61,35.43)
Congo	18.31 (8.69,32.05)	15.38 (6.84,28.07)	21.27 (9.93,37.18)
Austria	18.12 (7.92,35.42)	20.55 (8.71,40.96)	15.52 (6.71,28.93)
Bulgaria	18.03 (7.77,34.52)	21.56 (9.16,42.08)	14.28 (5.9,28.22)
Honduras	17.96 (8.14,30.17)	16.99 (7.55,31.24)	18.97 (8.46,33.74)
Guatemala	17.79 (7.64,33.15)	18.78 (8.07,36)	16.78 (6.78,30.97)
Hungary	17.67 (7.46,34.37)	21.45 (9.35,41.82)	13.69 (5.31,26.65)
Belgium	17.57 (7.59,34.22)	16.87 (7.29,33.07)	18.31 (7.97,35.5)

Location	BothNo. (95%UI)	MaleNo. (95%UI)	FemaleNo. (95%UI)
Burkina Faso	17.42 (8.44,31.1)	16.09 (7.7,29.54)	18.74 (8.57,35.23)
Papua New Guinea	17.07 (8.05,28.78)	13.88 (6.29,25.17)	20.6 (9.78,34.5)
Algeria	16.78 (7.54,32.14)	14.95 (6.65,27.81)	18.71 (8.56,35.98)
Sierra Leone	16.69 (7.48,29.47)	18.03 (8.04,32.49)	15.36 (6.93,27.56)
Nigeria	16.45 (7.35,29.73)	18.48 (7.98,35.06)	14.48 (6.47,27.8)
Serbia	16.19 (6.76,31.05)	16.73 (7.12,31.66)	15.59 (6.52,28.92)
Gabon	15.82 (7.1,27.51)	13.67 (6.14,25.29)	17.85 (8.04,30.57)
South Africa	15.82 (7.67,25.98)	14.32 (6.87,23.86)	17.35 (8.22,29.26)
Yemen	15.8 (7.33,28.97)	16.61 (7.28,30.32)	14.94 (6.6,27.13)
Albania	15.7 (6.56,29.61)	18.28 (7.58,34.01)	12.86 (5.47,24.18)
Czechia	15.64 (6.75,30.09)	19.75 (8.24,38.32)	11.31 (4.42,22.11)
Democratic People's Republic of Korea	15.58 (6.89,30.15)	17.66 (7.67,34.18)	13.4 (5.74,27.45)
Philippines	15.45 (7.29,27.71)	16.58 (7.65,29.96)	14.23 (6.57,25.82)
Israel	15.43 (6.69,29.32)	16.92 (7.3,32.65)	13.85 (5.95,26)
Belarus	15.2 (6.09,28.91)	17.23 (6.77,33.2)	13.04 (5.26,25.37)
Italy	14.64 (6.18,28.9)	17.5 (7.4,34.7)	11.61 (4.61,22.07)
Japan	14.61 (6.12,27.89)	14.62 (6.19,28.5)	14.6 (5.78,28.95)
Russian Federation	14.61 (6.27,27.34)	15.74 (6.67,30.1)	13.41 (5.48,25.43)
Burundi	14.55 (6.34,25.79)	12.34 (5.25,22.63)	16.71 (7.11,32.11)
Sao Tome and Principe	14.17 (6.4,24.87)	13.03 (5.93,23.1)	15.33 (6.59,28.54)
Mauritius	14.03 (6.21,24.43)	13.37 (5.75,25.63)	14.71 (6.64,25.92)
Morocco	14 (6.28,25.63)	13.19 (5.72,24.32)	14.85 (6.7,27.21)
Cameroon	13.92 (6.73,23.15)	13.98 (6.47,24.17)	13.85 (6.8,24.18)
Malaysia	13.76 (6.08,25.71)	13.64 (5.92,25.77)	13.89 (6.19,27.07)
Benin	13.72 (6.37,24.86)	15.22 (7.12,27.88)	12.19 (5.12,22.87)
Thailand	13.72 (6.17,26.23)	14.39 (6.16,26.57)	13.02 (5.59,24.45)
Ukraine	13.6 (5.65,25.94)	16.85 (7.11,33.55)	10.16 (3.9,20.55)
Republic of Korea	13.36 (5.78,25.38)	15.23 (6.33,29.16)	11.4 (4.88,22.04)
Namibia	13.03 (5.89,21.92)	11.35 (4.85,18.97)	14.7 (6.44,26.03)
Sudan	12.94 (5.81,22.69)	12.15 (5.35,22.29)	13.79 (5.95,23.53)
Seychelles	12.81 (5.67,23.39)	12.7 (5.4,24.33)	12.93 (5.42,23.95)
Timor-Leste	12.81 (5.83,22.48)	13.56 (6.49,24.68)	12.02 (5.08,21.88)
Uzbekistan	12.69 (5.49,23.87)	11.92 (5.08,22.83)	13.51 (5.93,24.86)
Eritrea	12.67 (5.87,22.4)	12.7 (5.95,22.13)	12.64 (5.46,22.86)
Democratic Republic of the Congo	12.65 (5.73,23.28)	10.11 (4.51,17.91)	15.28 (6.79,29.26)
Vanuatu	12.52 (5.67,22.48)	10.82 (4.56,18.28)	14.35 (6.14,30.46)
Estonia	12.35 (5.21,24.29)	14.6 (6.06,29.11)	9.99 (3.9,18.81)
Slovakia	12.34 (5.18,24.52)	16.33 (6.76,32.6)	8.14 (3.07,16.22)
Liberia	12.03 (5.34,21.85)	10.78 (4.92,19.76)	13.32 (5.87,25.5)
Singapore	11.99 (5.14,24.44)	14.63 (6.14,28.56)	9.24 (3.91,19.21)
Georgia	11.94 (5.27,22.81)	14.08 (6.13,26.89)	9.58 (4.08,18.87)
Afghanistan	11.84 (5.4,21.24)	10.21 (4.51,19.03)	13.59 (6.22,23.67)

Location	BothNo. (95%UI)	MaleNo. (95%UI)	FemaleNo. (95%UI)
Latvia	11.66 (4.79,22.79)	14.6 (5.8,27.96)	8.53 (3.62,16.85)
Kenya	11.19 (4.91,20.21)	9.71 (4.26,17.71)	12.72 (5.63,24.12)
Republic of Moldova	10.93 (4.68,21.12)	12.29 (5.01,23.82)	9.48 (3.82,19.56)
Lesotho	10.9 (5.32,18.41)	8.29 (3.91,14.72)	13.54 (6.02,23.74)
Azerbaijan	10.79 (4.58,20.2)	11.42 (4.71,21.8)	10.07 (4.31,19.08)
Guinea	10.75 (4.92,19.4)	10.6 (4.47,19.17)	10.91 (5.2,20.01)
Armenia	10.66 (4.47,20.69)	11.6 (4.84,22.49)	9.61 (3.74,19.3)
Lithuania	10.23 (4.23,19.67)	13.33 (5.52,25.3)	6.98 (2.73,14.07)
Togo	10.18 (4.59,19.76)	10.31 (4.63,19.68)	10.04 (4.59,19.22)
Kyrgyzstan	10.04 (4.22,19.19)	10.69 (4.5,20.2)	9.36 (3.79,18.25)
China	9.99 (4.09,19.57)	12.27 (5.01,24)	7.37 (3.03,14.54)
Chad	9.62 (4.47,16.73)	11.75 (5.17,20.9)	7.45 (3.28,13.53)
Guinea-Bissau	9.6 (4.26,17.01)	10.47 (4.51,18.49)	8.72 (3.77,17.38)
Cabo Verde	9.41 (4.03,17.86)	8.73 (3.57,16.78)	10.12 (4.18,19.53)
Solomon Islands	9.31 (4.26,16.05)	8.34 (3.73,14.68)	10.37 (4.8,18.32)
Brunei Darussalam	9.12 (3.81,17.72)	10.93 (4.72,20.7)	7.13 (2.86,14.67)
Turkmenistan	8.98 (3.84,16.65)	9.2 (4.01,17.34)	8.74 (3.53,16.4)
Zimbabwe	8.73 (3.9,14.98)	5.4 (2.29,9.66)	12.05 (5.23,21.12)
Maldives	8.67 (3.91,16.04)	9.54 (4.03,17.86)	7.74 (3.34,13.97)
Sri Lanka	8.31 (3.75,14.86)	7.58 (3.37,13.94)	9.06 (4.07,16.95)
Mongolia	8.05 (3.3,15.2)	7.85 (3.23,15.33)	8.27 (3.31,15.54)
Myanmar	8.04 (3.77,13.28)	7.32 (3.43,12.21)	8.79 (4.02,15.21)
C te d'Ivoire	7.76 (3.39,14.49)	7.48 (3.32,13.75)	8.05 (3.42,15.22)
Indonesia	7.67 (3.69,13.27)	7.49 (3.44,13.35)	7.86 (3.82,13.77)
Tajikistan	7.65 (3.16,14.42)	7.53 (3,14.5)	7.78 (3.24,15.41)
Ethiopia	7.54 (3.45,12.97)	6.68 (2.81,12.1)	8.44 (3.74,15.61)
Niger	7.48 (3.42,13.47)	7.39 (3.11,13.59)	7.58 (3.39,14.12)
Senegal	6.96 (3.2,12.73)	5.15 (2.27,9.56)	8.9 (4.01,17.05)
Kazakhstan	6.9 (2.9,13.36)	6.61 (2.9,12.92)	7.2 (2.97,14.07)
Lao People's Democratic Republic	6.72 (2.95,12.05)	6.14 (2.68,11.01)	7.33 (3.22,14.13)
Ghana	6.5 (3.02,11.53)	5.48 (2.37,10.37)	7.56 (3.2,13.65)
Mali	6.07 (2.84,11.31)	5.94 (2.67,10.75)	6.22 (2.57,13.23)
Bhutan	5.72 (2.46,10.23)	4.61 (1.95,8.44)	6.84 (2.81,12)
Nepal	5.33 (2.47,9.21)	5.54 (2.45,9.99)	5.11 (2.15,9.61)
Viet Nam	4.84 (2.17,8.91)	5.47 (2.41,10.77)	4.16 (1.8,7.7)
Gambia	4.72 (2.02,8.51)	4.03 (1.79,7.43)	5.42 (2.25,10.22)
India	4.54 (2,8.56)	4.84 (2.07,9.37)	4.21 (1.9,7.92)
Cambodia	3.54 (1.63,6.33)	3.58 (1.57,6.59)	3.5 (1.56,6.41)
Pakistan	3.54 (1.59,6.32)	3.61 (1.59,6.45)	3.46 (1.41,6.6)
Bangladesh	3.09 (1.25,5.97)	2.76 (1.19,5.23)	3.43 (1.31,6.69)

Supplementary Table S2 The distribution of mortality rate, DALYs rate, YLDs rate, and YLLs rate attributable to high BMI for childhood and adolescent asthma across different SDI regions in 2021.

Location	Sex	Death rate (per 100,000)	DALYs rate (per 100,000)	YLDs rate (per 100,000)	YLLs rate (per 100,000)
Global	Both	0.02 (0.01,0.03)	15.76 (7.31,28.26)	14.03 (6.35,25.83)	1.73 (0.85,2.71)
Global	Fema	0.02 (0.01,0.04)	15.12 (6.99,26.85)	13.17 (5.96,24.25)	1.95 (0.94,3.23)
Global	Male	0.02 (0.01,0.03)	16.36 (7.62,29.23)	14.83 (6.73,27.59)	1.53 (0.76,2.36)
High SDI	Both	0.02 (0.01,0.03)	43.47 (20.25,78.43)	41.99	1.48 (0.79,2.19)
High SDI	Fema	0.02 (0.01,0.03)	40.2 (18.46,72.3)	38.84 (17.66,70.5)	1.36 (0.73,2.02)
High SDI	Male	0.02 (0.01,0.03)	46.55 (21.92,84.34)	44.97	1.58 (0.84,2.4)
High-middle	Both	0.01 (0,0.01)	15.29 (6.62,28.59)	14.79 (6.28,27.99)	0.5 (0.26,0.76)
High-middle	Fema	0.01 (0,0.01)	13.24 (5.76,24.38)	12.75 (5.38,23.73)	0.49 (0.25,0.76)
High-middle	Male	0.01 (0,0.01)	17.15 (7.43,32.38)	16.65 (7.15,31.78)	0.5 (0.25,0.77)
Middle SDI	Both	0.02 (0.01,0.03)	14.64 (6.71,26.28)	13.32 (5.82,24.51)	1.32 (0.68,2.03)
Middle SDI	Fema	0.02 (0.01,0.03)	13.93 (6.26,25.05)	12.49 (5.45,23.05)	1.44 (0.72,2.29)
Middle SDI	Male	0.02 (0.01,0.02)	15.3 (6.91,28.02)	14.09 (6.03,26.19)	1.21 (0.62,1.91)
Low-middle	Both	0.02 (0.01,0.03)	10.05 (4.67,17.74)	8.51 (3.78,15.78)	1.54 (0.77,2.42)
Low-middle	Fema	0.02 (0.01,0.04)	9.98 (4.68,17.57)	8.21 (3.7,15.24)	1.77 (0.87,2.84)
Low-middle	Male	0.02 (0.01,0.03)	10.11 (4.62,18.05)	8.8 (3.86,16.31)	1.31 (0.65,2.06)
Low SDI	Both	0.04 (0.02,0.07)	13.84 (6.21,24.14)	10.59 (4.79,19.81)	3.25 (1.46,5.47)
Low SDI	Fema	0.05 (0.02,0.09)	14.29 (6.44,25.4)	10.52 (4.87,19.36)	3.77 (1.67,7.1)
Low SDI	Male	0.03 (0.01,0.06)	13.4 (6.01,23.39)	10.65 (4.78,20.11)	2.75 (1.24,4.6)

Supplementary Table S3 Concentration index of DALYs among children and adolescents with asthma caused by high BMI with 95% confidence intervals, 1990-2021.

	concentration index (95% CI)		
year	Both	Male	Women
1990	0.234 (0.212, 0.255)	0.267 (0.245, 0.29)	0.195 (0.173, 0.218)
1991	0.23 (0.208, 0.252)	0.263 (0.241, 0.285)	0.194 (0.171, 0.216)
1992	0.227 (0.205, 0.249)	0.258 (0.236, 0.28)	0.192 (0.169, 0.215)
1993	0.225 (0.204, 0.247)	0.256 (0.234, 0.278)	0.192 (0.169, 0.215)
1994	0.224 (0.202, 0.246)	0.254 (0.232, 0.276)	0.192 (0.169, 0.215)
1995	0.224 (0.202, 0.246)	0.252 (0.229, 0.274)	0.194 (0.17, 0.217)
1996	0.224 (0.202, 0.247)	0.251 (0.229, 0.273)	0.195 (0.172, 0.219)
1997	0.226 (0.204, 0.248)	0.252 (0.23, 0.274)	0.198 (0.175, 0.221)
1998	0.228 (0.206, 0.249)	0.253 (0.231, 0.275)	0.2 (0.177, 0.222)
1999	0.23 (0.208, 0.251)	0.254 (0.232, 0.276)	0.202 (0.18, 0.224)
2000	0.232 (0.21, 0.253)	0.256 (0.234, 0.277)	0.205 (0.183, 0.227)
2001	0.234 (0.213, 0.255)	0.256 (0.235, 0.278)	0.209 (0.187, 0.231)
2002	0.235 (0.214, 0.256)	0.257 (0.235, 0.278)	0.211 (0.19, 0.233)
2003	0.234 (0.213, 0.256)	0.255 (0.234, 0.277)	0.212 (0.19, 0.233)
2004	0.233 (0.212, 0.254)	0.252 (0.231, 0.274)	0.211 (0.19, 0.233)
2005	0.23 (0.209, 0.252)	0.248 (0.226, 0.269)	0.211 (0.19, 0.233)
2006	0.228 (0.207, 0.249)	0.243 (0.221, 0.265)	0.212 (0.191, 0.234)
2007	0.225 (0.204, 0.246)	0.237 (0.215, 0.258)	0.213 (0.192, 0.235)
2008	0.223 (0.201, 0.244)	0.23 (0.208, 0.252)	0.215 (0.193, 0.236)
2009	0.222 (0.2, 0.243)	0.227 (0.204, 0.249)	0.217 (0.195, 0.238)
2010	0.221 (0.2, 0.243)	0.225 (0.203, 0.247)	0.218 (0.197, 0.239)
2011	0.223 (0.202, 0.244)	0.227 (0.205, 0.249)	0.219 (0.198, 0.24)
2012	0.227 (0.206, 0.248)	0.232 (0.209, 0.254)	0.223 (0.202, 0.244)
2013	0.232 (0.211, 0.253)	0.237 (0.215, 0.259)	0.228 (0.207, 0.248)
2014	0.236 (0.215, 0.257)	0.242 (0.22, 0.264)	0.231 (0.21, 0.252)
2015	0.239 (0.218, 0.26)	0.244 (0.222, 0.266)	0.233 (0.213, 0.254)
2016	0.239 (0.218, 0.26)	0.244 (0.222, 0.266)	0.234 (0.213, 0.255)
2017	0.24 (0.219, 0.26)	0.245 (0.223, 0.267)	0.234 (0.213, 0.255)
2018	0.24 (0.219, 0.261)	0.245 (0.223, 0.267)	0.235 (0.214, 0.256)
2019	0.24 (0.219, 0.261)	0.244 (0.223, 0.266)	0.235 (0.214, 0.256)
2020	0.236 (0.215, 0.257)	0.239 (0.217, 0.261)	0.232 (0.211, 0.253)
2021	0.235 (0.214, 0.256)	0.236 (0.214, 0.258)	0.234 (0.213, 0.255)

Supplementary Table S4 The age-standardized number and rate of deaths from asthma in childhood and adolescence attributable to high BMI (1990 and 2021)

		1990		2021	
Location	Sex	Number No.(95%CI)	ASMR(per 100,000) No.(95%CI)	Number No.(95%CI)	ASMR(per 100,000) No.(95%CI)
Global	Both	194.71 (89.02,324.83)	0.03 (0.02,0.06)	142.81 (69.38,227.89)	0.02 (0.01,0.03)
Global	Female	97.92(37.43,174.71)	0.03 (0.01,0.06)	77.81 (37.17,129.29)	0.02 (0.01,0.04)
Global	Male	96.79 (44.51,155.41)	0.03 (0.01,0.05)	65.00 (31.30,104.60)	0.02 (0.01,0.03)
High SDI	Both	16.23 (8.07,25.46)	0.03 (0.01,0.04)	10.75 (5.73,16.02)	0.02 (0.01,0.03)
High SDI	Female	6.22 (3.10,9.90)	0.02 (0.01,0.03)	4.82 (2.59,7.23)	0.02 (0.01,0.03)
High SDI	Male	10.01 (4.91,15.71)	0.03 (0.02,0.05)	5.94 (3.12,8.93)	0.02 (0.01,0.03)
High-middle SDI	Both	13.05 (6.21,21.20)	0.01 (0.01,0.02)	4.70 (2.37,7.35)	0.01 (0,0.01)
High-middle SDI	Female	5.42(2.39,9.56)	0.01 (0.01,0.02)	2.22 (1.13,3.60)	0.01 (0,0.01)
High-middle SDI	Male	7.63 (3.57,12.49)	0.02 (0.01,0.03)	2.48 (1.21,4.01)	0.01 (0,0.01)
Middle SDI	Both	50.66 (23.68,84.56)	0.03 (0.01,0.04)	30.95 (15.76,48.97)	0.02 (0.01,0.03)
Middle SDI	Female	25.56 (10.66,45.12)	0.03 (0.01,0.05)	16.14 (7.98,26.64)	0.02 (0.01,0.03)
Middle SDI	Male	25.10 (11.65,40.55)	0.03 (0.01,0.04)	14.81 (7.43,23.70)	0.02 (0.01,0.02)
Low-middle SDI	Both	62.05 (27.70,104.52)	0.04 (0.02,0.07)	36.67 (17.87,58.49)	0.02 (0.01,0.03)
Low-middle SDI	Female	32.15 (11.67,59.41)	0.04 (0.02,0.08)	20.56 (9.70,34.30)	0.02 (0.01,0.04)
Low-middle SDI	Male	29.90 (13.62,50.04)	0.04 (0.02,0.06)	16.12 (7.61,26.72)	0.02 (0.01,0.03)
Low SDI	Both	52.45 (20.88,95.39)	0.06 (0.03,0.12)	59.47 (26.29104.39)	0.04 (0.02,0.07)
Low SDI	Female	28.44 (8.80,55.44)	0.07 (0.02,0.14)	33.94 (14.83,65.61)	0.05 (0.02,0.09)
Low SDI	Male	24.01 (10.38,41.20)	0.06 (0.02,0.10)	25.53 (11.17,43.87)	0.03 (0.01,0.06)

Supplementary Table S5 The age-standardized number and rate of DALYs from asthma in childhood and adolescence attributable to high BMI (1990 and 2021)

		1990		2021	
Location	Sex	Number<br>No.(95%CI)	ASDR(per 100,000)<br>No.(95%CI)	Number<br>No.(95%CI)	ASDR(per 100,000)<br>No.(95%CI)
Global	Both	74802.23 (33793.40,137020.62)	12.99 (5.87,23.81)	104881.24 (47056.32,197319.50)	15.80 (7.09,29.70)
Global	Female	35002.89 (15545.27,64528.45)	12.51 (5.56,23.07)	48516.82 (21826.73,90502.25)	15.11 (6.80,28.17)
Global	Male	39799.34 (17880.38,73196.10)	13.43 (6.03,24.72)	56364.42 (25256.55,105495.27)	16.44 (7.36,30.75)
High SDI	Both	18128.95 (7956.11,34362.05)	28.92 (12.69,54.84)	25130.61 (11165.18,47642.56)	42.93 (19.03,81.41)
High SDI	Female	7657.67 (3225.61,14912.95)	25.05 (10.55,48.82)	11200.68 (4925.64,21021.51)	39.25 (17.25,73.73)
High SDI	Male	10471.28 (4517.57,20287.44)	32.60 (14.06,63.18)	13929.93 (6106.26,26169.59)	46.43 (2031,87.28)
High-middle SDI	Both	8762.31 (3744.35,16556.49)	9.52 (4.07,17.99)	11705.37 (4828.01,23039.99)	15.28 (6.29,30.05)
High-middle SDI	Female	3646.89(1555.36,6943.25)	8.16 (3.48,15.54)	4798.35 (2000.11,9444.27)	13.15 (5.47,25.85)
High-middle SDI	Male	5115.42 (2196.96,9646.94)	10.80 (4.64,20.36)	6907.02 (2832.37,13612.12)	17.22 (7.05,33.93)
Middle SDI	Both	21436.37 (9456.45,39424.06)	11.12 (4.90,20.46)	27709.92 (12060.17,52627.83)	14.70 (6.40,27.90)
Middle SDI	Female	10189.43 (4498.84,19077.80)	10.89 (4.81,20.39)	12603.42 (5572.58,23793.54)	13.93 (6.16,26.26)
Middle SDI	Male	11264.94 (4875.76,20609.22)	11.33 (4.91,20.76)	15106.50 (6451.24,29015.92)	15.41 (6.58,29.60)
Low-middle SDI	Both	16054.57 (7358.12,28934.66)	10.27 (4.70,18.52)	19404.52 (8854.69,35734.33)	10.11 (4.61,18.61)
Low-middle SDI	Female	7876.01 (3494.72,14375.57)	10.45 (4.64,19.10)	9321.38 (4287.99,17117.99)	10.00 (4.60,18.36)
Low-middle SDI	Male	8178.56 (3662.07,14757.06)	10.09 (4.51,18.26)	10083.15 (4490.47,18792.83)	10.20 (4.54,19.01)
Low SDI	Both	10323.69 (4613.09,18332.09)	13.12 (5.87,23.34)	20796.86 (9415.73,36743.61)	13.69 (6.20,24.20)
Low SDI	Female	5586.55 (2380.25,10293.99)	14.65 (6.30,27.01)	10530.15 (4728.09,18790.95)	14.19 (6.38,25.33)
Low SDI	Male	4737.13 (2152.96,8413.59)	11.63 (5.27,20.69)	10266.72 (4729.53,18165.65)	13.20 (6.07,23.38)

Supplementary Table S6 Projection of DALYs Attributable to High BMI for Childhood and Adolescent Asthma by 2036.

Year	Both No.(95%CI)	Male No.(95%CI)	Female No.(95%CI)
2022	15.92(15.63-16.69)	16.54(15.86-17.04)	15.19(14.59-15.74)
2023	16.04(15.46-17.09)	16.52(15.45-17.37)	15.18(14.25-16.07)
2024	16.16(15.26-17.54)	16.45(15.07-17.79)	15.16(13.9-16.47)
2025	16.28(15.02-18.02)	16.41(14.74-18.27)	15.16(13.55-16.91)
2026	16.4(14.75-18.53)	16.43(14.42-18.78)	15.19(13.17-17.37)
2027	16.52(14.45-19.07)	16.51(14.08-19.28)	15.23(12.77-17.85)
2028	16.64(14.13-19.63)	16.6(13.7-19.78)	15.27(12.34-18.35)
2029	16.76(13.78-20.22)	16.68(13.27-20.29)	15.31(11.88-18.87)
2030	16.88(13.41-20.84)	16.74(12.8-20.82)	15.34(11.39-19.41)
2031	17(13.01-21.48)	16.78(12.3-21.38)	15.37(10.88-19.98)
2032	17.12(12.59-22.14)	16.81(11.8-21.97)	15.4(10.35-20.58)
2033	17.24(12.15-22.82)	16.84(11.28-22.59)	15.43(9.8-21.19)
2034	17.36(11.69-23.52)	16.88(10.75-23.23)	15.47(9.23-21.83)
2035	17.48(15.83-16.01)	16.94(16.46-16.63)	15.5(15.09-15.28)
2036	17.6(15.75-16.32)	16.99(16.23-16.81)	15.53(14.9-15.47)

Supplementary Figure F1: the burden of asthma attributable to high BMI in 2021 varied across age groups and across sex groups

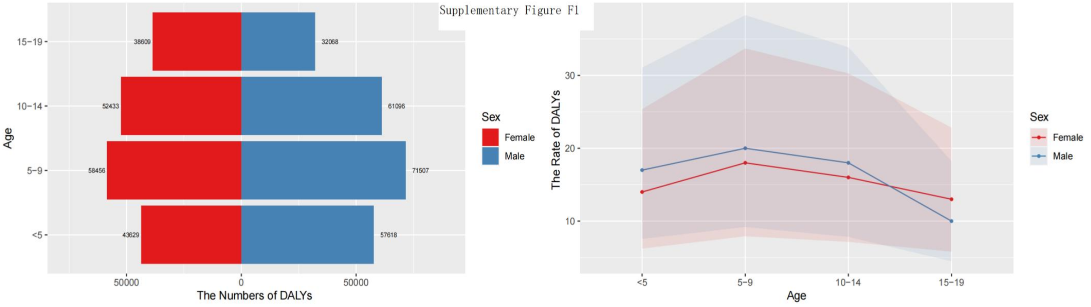

Supplementary Figure F2: Correlation analysis between ASDR(per 100,000 population) and SDI in 204 countries

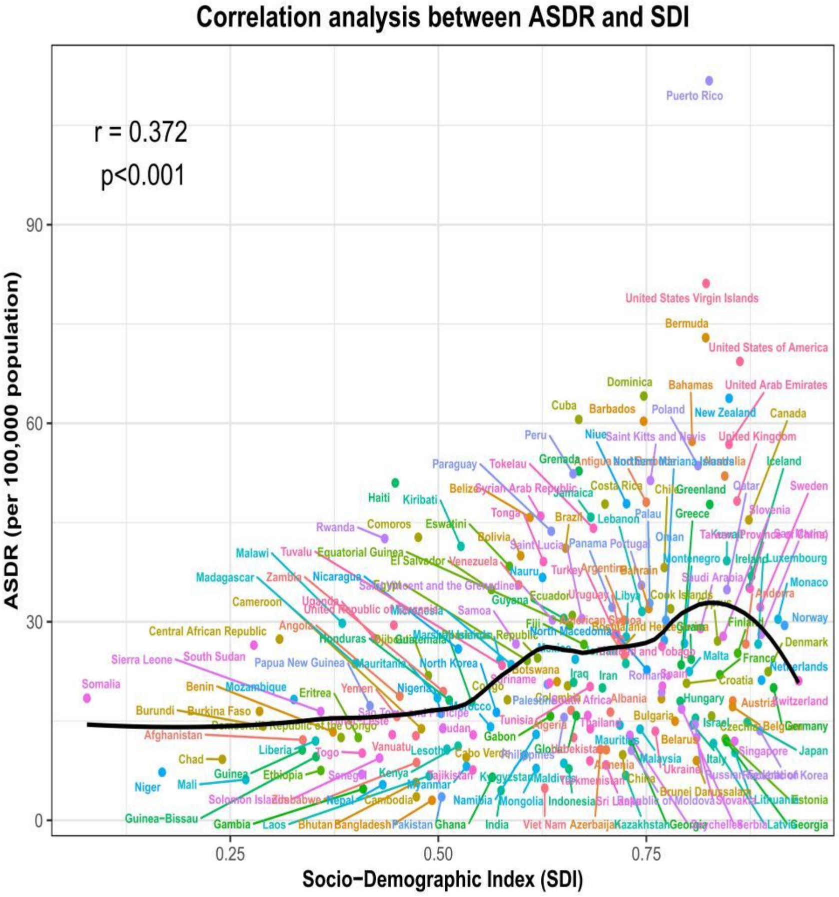

Supplementary Figure F3: Correlation analysis between ASDMR(per 100,000 population) and SDI in 204 countries

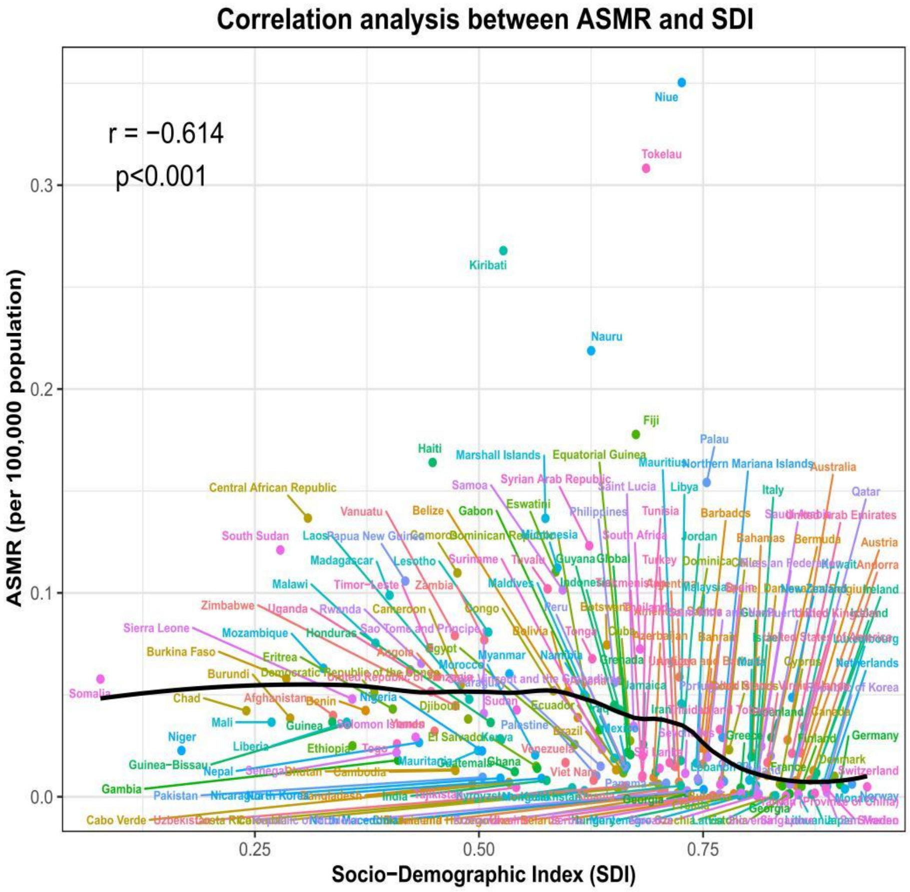

Supplementary Figure F4: Cross-country inequality analysis of asthma attributable to high BMI

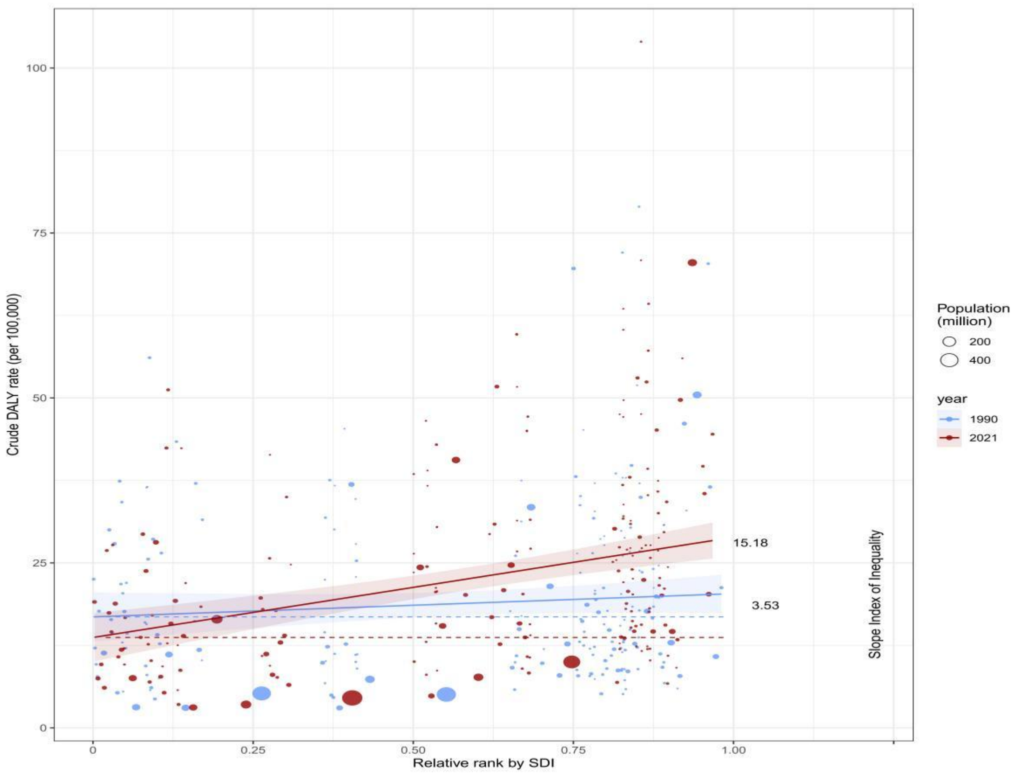

Supplementary Figure F5: Cross-country inequality analysis of asthma attributable to high BMI

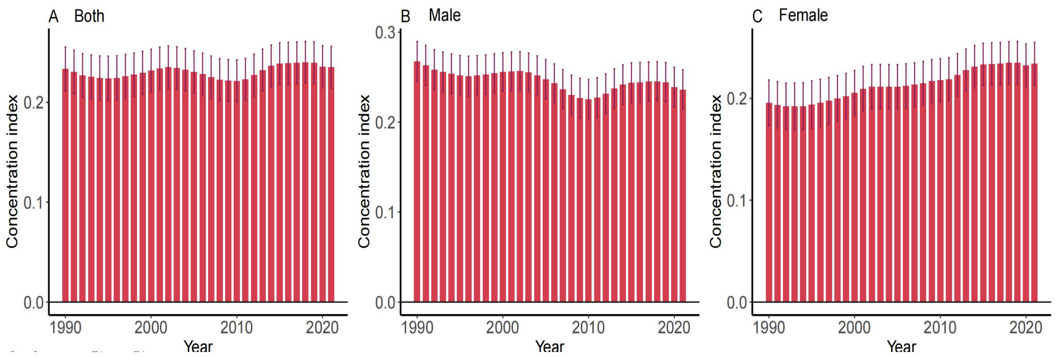

Supplementary Figure F6 Projection of DALYs Attributable to High BMI for Childhood and Adolescent Asthma by 2036.

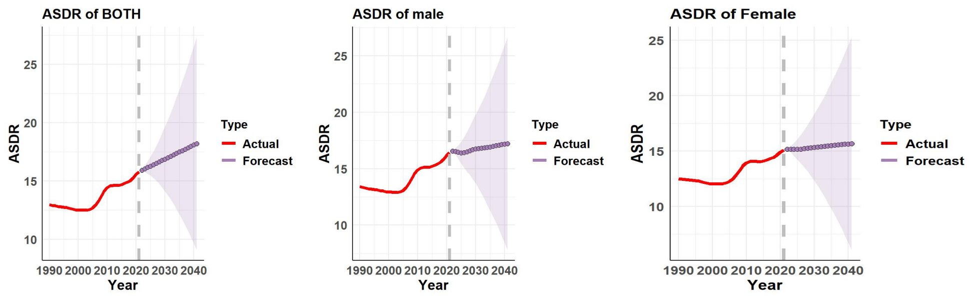

Supplementary Figure F7 Prediction analysis of asthma burden attributable to high BMI： the age-standardized DALYs rate and deaths rate per 100,000

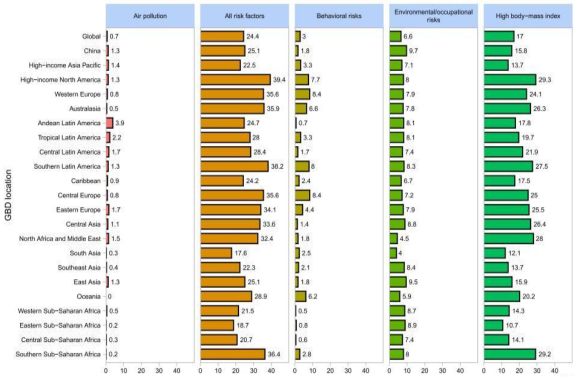

7A

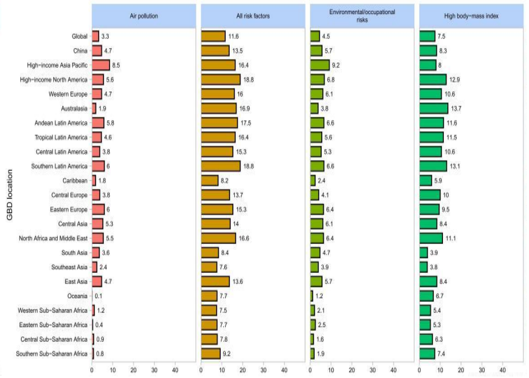

7B

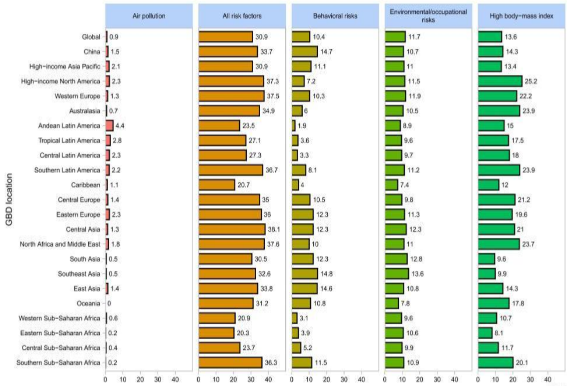

7C

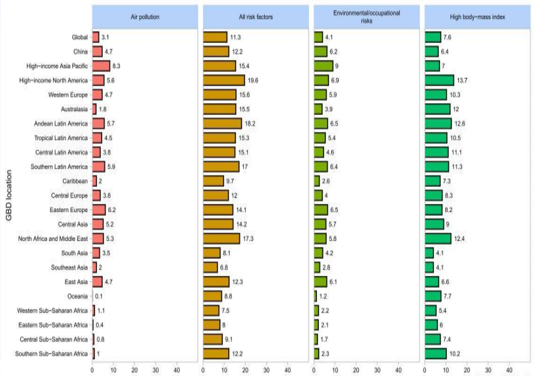

7D

Supplementary Figure F7 A.Female -all ages B. Male- <20 years C.Male-all ages D.Female-<20 years .DALYs attributable to risk factors (%) among all ages and <20 years between male and female
